# Changes in transverse dimensions in growing patients treated with AMCOP® and electromyographic assessment with Teethan®

**DOI:** 10.3389/fdmed.2026.1738007

**Published:** 2026-03-12

**Authors:** Laura Ferrante, Gianna Dipalma, Filippo Cardarelli, Angela Di Noia, Grazia Marinelli, Antonio Di Lorenzo, Francesco Inchingolo, Daniela Di Venere, Andrea Palermo, Angelo Michele Inchingolo, Alessio Danilo Inchingolo

**Affiliations:** 1 Interdisciplinary Department of Medicine, University of Bari ‘Aldo Moro’, Bari, Italy; 2 Department of Biomedical, Surgical and Dental Sciences, Milan University, Milan, Italy; 3 Interdisciplinary Department of Medicine, University of Salento, Lecce, Italy

**Keywords:** AMCOP, Deltadent, digital models, elastodontics, pediatric orthodontics, surface electromyography, Teethan, transverse maxillary deficiency

## Abstract

**Aim:**

To retrospectively evaluate transverse maxillary changes and neuromuscular balance in growing patients treated with AMCOP® elastodontic appliances by combining three-dimensional digital model analysis and surface electromyography (sEMG).

**Materials and methods:**

This monocentric retrospective case series included 12 children (8 females, 4 males; aged 4–7 years) treated for transverse maxillary deficiency with AMCOP® Integral or Class III (TC) devices for 10–18 months. Transverse widths were measured on digital models (Deltadent®) at baseline (T0) and post-treatment (T1) at the intercanine, inter–second deciduous molar (inter-5), and intermediate or first molar regions when erupted. Neuromuscular function was assessed using surface electromyography (Teethan®), evaluating symmetry indices (POC TA/MM), barycenter (BAR), torsion (TORS), asymmetry (ASIM), and impact index (IMP).

**Results:**

All patients showed transverse maxillary expansion. Intercanine increases ranged from +0.5 to +4.9 mm, inter-5 from +1.15 to +6.26 mm, and intermolar measurements (available in 6 patients) from +1.83 to +4.87 mm. sEMG recordings demonstrated improvement in neuromuscular coordination in most cases, including normalization or improvement of BAR, reduction of TORS and ASIM values, and enhancement of IMP toward physiological ranges. No adverse events were recorded.

**Conclusions:**

Within the limitations of this retrospective case series, AMCOP® elastodontic therapy was associated with clinically relevant transverse maxillary widening and favorable neuromuscular rebalancing. These preliminary findings support elastodontic functional interception as a conservative approach for early transverse deficiency, although controlled comparative studies are required to confirm treatment efficacy.

## 1 Introduction

The transverse development of the maxilla is a key determinant in establishing proper craniofacial harmony, occlusal balance, and airway function. This process originates early during embryogenesis, when the maxillary and palatine shelves fuse between the 7th and 12th weeks of gestation to form the hard palate and midpalatal suture (1–5). Following birth, this suture undergoes progressive appositional growth, largely modulated by functional stimuli such as nasal breathing, tongue posture, swallowing, and masticatory loading. The dynamic interplay between skeletal structures and soft tissue function ensures a coordinated increase in the width of the maxilla during early childhood. During the active growth period, particularly between 6 and 12 years of age, the midpalatal suture remains patent, allowing for both spontaneous and therapeutically induced expansion (5–11). This period represents a window of opportunity for orthopedic and functional interventions aimed at redirecting craniofacial growth patterns before skeletal maturation limits adaptive potential. Transverse maxillary discrepancies are defined as conditions in which the maxillary arch width is insufficient relative to the mandibular arch. A well-balanced transverse relationship typically exhibits a maxillary intermolar width 2–3 mm greater than that of the mandible, allowing for optimal occlusal interdigitation (McNamara, 2000). When this proportion is reversed, crossbites and arch constrictions emerge, often classified as mild (<3 mm), moderate (3–5 mm), or severe (>5 mm), depending on the magnitude of discrepancy and the patient's developmental stage (12–18). The etiology of transverse deficiency is multifactorial. Genetic predispositions may be compounded by environmental and functional influences such as chronic mouth breathing, upper airway obstruction (adenoid hypertrophy, allergic rhinitis), atypical swallowing, altered tongue posture, and parafunctional habits including thumb sucking or prolonged pacifier use (19–22). These factors act synergistically to disturb the equilibrium between the maxillary skeletal base and the neuromuscular environment, producing both morphological and functional imbalances (23–29). Epidemiological data indicate that transverse discrepancies affect approximately 8%–22% of children in the early mixed dentition, making them one of the most frequent developmental alterations encountered in pediatric orthodontics. If left untreated, maxillary constriction may contribute to a cascade of secondary problems, posterior crossbite, dental compensation, midline deviation, asymmetric mandibular growth, temporomandibular dysfunctions (TMD), and impaired masticatory efficiency (30–36). Furthermore, the close anatomical and functional interrelationship between the maxilla, nasal cavity, and cranial base implies that transverse deficiency can also influence respiratory patterns and even global postural balance.

The timing of intervention plays a decisive role in the orthopedic management of transverse maxillary deficiencies. In the mixed dentition stage, the midpalatal suture is less mature and more responsive to orthopedic stimuli; therefore, a substantial part of the expansion results from true skeletal distraction rather than mere dento-alveolar remodeling. Comparative data between children and adults show that in pediatric patients, approximately half of the expansion occurs at the skeletal level through palatal separation, whereas in adults the skeletal component is markedly reduced and dental tipping predominates (37–44). Early treatment also benefits from residual growth potential, which facilitates the adaptation of soft tissues and contributes to long-term stability (45–53). Clinical series and longitudinal observations have demonstrated that when expansion is performed during early growth, there are no significant adverse vertical effects or increases in mandibular plane inclination, provided that the expansion protocol is well controlled (54–62). Moreover, early interceptive expansion contributes to an increase in arch perimeter, reducing the need for extractions to resolve mild or moderate crowding. Hence, intervening during the period of high sutural plasticity allows the clinician to achieve greater orthopedic effectiveness with minimal invasiveness, promoting harmonious development of the maxillary complex and the surrounding functional matrices.

Conventional orthopedic expansion techniques are primarily mechanical in nature and aim to separate the midpalatal suture through the application of transverse forces. Rapid palatal expansion (RPE) devices, such as Hyrax and Haas expanders, are well-established tools capable of producing skeletal expansion through a combination of sutural and dental effects. Similarly, slow expansion appliances like the quad-helix and W-arch deliver lighter forces over a longer period, promoting gradual adaptation of the supporting tissues. Although these appliances have demonstrated consistent clinical success in increasing maxillary width, their action is largely limited to the orthopedic domain. They tend to disregard the underlying neuromuscular dysfunctions, altered tongue posture, unbalanced masticatory muscle activity, or dysfunctional swallowing, that frequently accompany skeletal constriction (63–70). Consequently, relapse may occur if the etiologic functional disturbances remain uncorrected. Fixed expansion appliances may also induce unwanted dental tipping, root resorption, and patient discomfort, particularly in younger subjects. Hygiene difficulties and interference with phonation or feeding further limit their acceptance in pediatric populations. These considerations have motivated the search for alternative modalities that can achieve not only skeletal widening but also the restoration of normal orofacial function (71–76).

Recent decades have witnessed the emergence of functional orthopedic philosophies that prioritize the integration of skeletal, muscular, and postural components in the correction of maxillary discrepancies. The AMCOP® (Apparecchi Modulari di Contenzione Ortopedica Personalizzati) system represents a significant innovation in this context. Designed as a modular and elastodontic appliance, AMCOP® combines the mechanical action necessary for orthopedic remodeling with the re-education of the orofacial musculature and proprioceptive control. Constructed from elastic medical-grade materials, the device exerts light, continuous forces that guide growth along physiological trajectories rather than imposing rigid displacement.ts modular design allows customization according to each patient's developmental stage and functional needs (77–84). The appliance promotes correct tongue positioning, enhances nasal respiration, balances mandibular posture, and facilitates neuromuscular reprogramming of swallowing and masticatory patterns. Unlike fixed expanders, AMCOP® acts through neuromuscular activation, stimulating the natural plasticity of craniofacial structures (85–90). By harmonizing the relationship between bone, muscle, and function, it aims to produce more stable and physiologically integrated results. Compared with traditional expanders such as Hyrax or Haas, AMCOP® induces a gradual orthopedic-functional expansion supported by:.

- a functional masticatory plane that decompresses the transverse corridors;
- a more centered and repetitive lingual thrust during swallowing, breathing, and phonation;
- neuromuscular retraining that reduces dysfunctional patterns such as anterior tongue interposition.

This approach is less invasive, requires no daily screw activation, and seeks to integrate skeletal, dental, and muscular adaptation within a unified functional framework (91–99).

Craniofacial growth cannot be dissociated from the neuromuscular environment that shapes and sustains it. The coordination of the masticatory muscles, tongue, and cervical musculature forms an integrated system influencing not only occlusion but also postural stability. The concept of functional equilibrium, introduced by Moss within the framework of the functional matrix theory, underscores the principle that skeletal morphology adapts to functional demands. Altered muscle tone or asymmetrical activation patterns, often present in children with transverse deficiencies, may induce postural compensations extending from the stomatognathic system to the cervical and thoracic regions (100–103). Therefore, functional evaluation is essential in both diagnosis and treatment planning. The incorporation of digital technologies such as Teethan® provides objective and quantitative assessment of neuromuscular activity through surface electromyography (sEMG). This system allows clinicians to monitor the coordination of masseter and temporalis muscles during occlusal function, identify asymmetries, and evaluate improvements following therapeutic intervention. In the context of AMCOP® therapy, Teethan® analysis contributes to verifying neuromuscular rebalancing and supports the concept that orthopedic correction should coincide with muscular symmetry and postural improvement (104–109).

Given the high prevalence of transverse maxillary deficiencies in early mixed dentition and their close association with functional alterations such as low tongue posture and oral breathing, an interceptive therapeutic approach addressing both skeletal and neuromuscular aspects is essential. The present study aims to evaluate the efficacy of AMCOP® elastodontic therapy in correcting transverse maxillary deficiencies in growing children aged 4–7 years. The treatment duration ranged from 10 to 18 months, corresponding to an optimal period of skeletal plasticity (104). All patients included in this case series were treated at the Department of Orthodontics, Policlinico of Bari (Italy), following ethical guidelines and with informed parental consent. Pre- and post-treatment digital measurements were performed to quantify the transverse skeletal changes using Deltadent® software (Deltadent S.r.l., Italy), while functional outcomes were supported by neuromuscular analyses obtained with Teethan® sEMG evaluation (110–113). By presenting twelve well-documented clinical cases, this research contributes original data to the field of pediatric orthodontics and underscores the potential of AMCOP® functional elastodontic therapy to harmonize craniofacial growth through physiologic transverse expansion. Ultimately, the goal of this work is to highlight the capacity of AMCOP® devices to promote balanced transverse development, restore functional harmony within the stomatognathic system, and prevent future relapses by addressing the underlying causes of maxillary constriction rather than its morphological consequences (114–116).

## 2 Materials and methods

### 2.1 Study design and setting

This was a monocentric, observational case series with retrospective data analysis conducted at the Department of Dentistry and Orthodontics, University Polyclinic of Bari (Italy). The study focused on growing patients with transverse maxillary deficiency treated with functional elastodontic appliances.

The study was conducted in accordance with the Declaration of Helsinki and approved by the Ethics Committee of the Policlinico of Bari (Protocol No. 971, Prot. 2427/CEL., approved on 1 October 2025, *U.O. di Odontostomatologia; Principal Investigator: Prof. F. Inchingolo*).

Written informed consent was obtained from all parents or legal guardians before data collection, image publication, and clinical participation.

### 2.2 Patient identification and case selection (retrospective database query)

Eligible cases were identified retrospectively by querying the institutional clinical database of the Department of Dentistry and Orthodontics, University Polyclinic of Bari, for growing patients treated with AMCOP® elastodontic appliances for transverse maxillary deficiency between January 2022 and december 2024. The search was performed using treatment logs and digital model archives (Deltadent®), cross-checked with Teethan® sEMG records when available. For each potentially eligible patient, the presence of baseline (T0) and end-of-treatment (T1) digital models and the completion of active transverse therapy (minimum 10 months) were verified. Only subjects meeting all inclusion/exclusion criteria and having analyzable T0–T1 records were included in the final case series.

### 2.3 Inclusion and exclusion criteria

Inclusion criteria:

- Age between 4 and 7 years at the start of treatment (late deciduous or early mixed dentition).
- Transverse maxillary deficiency documented clinically and on digital models, including maxillary constriction, unilateral or bilateral posterior crossbite, or posterior reverse bite.
- No previous orthopedic or orthodontic treatment aimed at transverse correction.
- Availability of reliable pre- and post-treatment digital models (T0 and T1).
- Additional inclusion criterion (data completeness): Availability of complete and analyzable T0 and T1 digital models; sEMG availability was required when the neuromuscular outcomes were reported (Teethan® indices).

Exclusion criteria:

- Cleft palate or other craniofacial malformations.
- Systemic or craniofacial growth disorders affecting bone metabolism or facial development.
- Severe airway obstruction or medical conditions incompatible with follow-up.
- Low compliance or anticipated inability to complete at least 10 months of active therapy.

### 2.4 Sample characteristics

**Figure 1:**
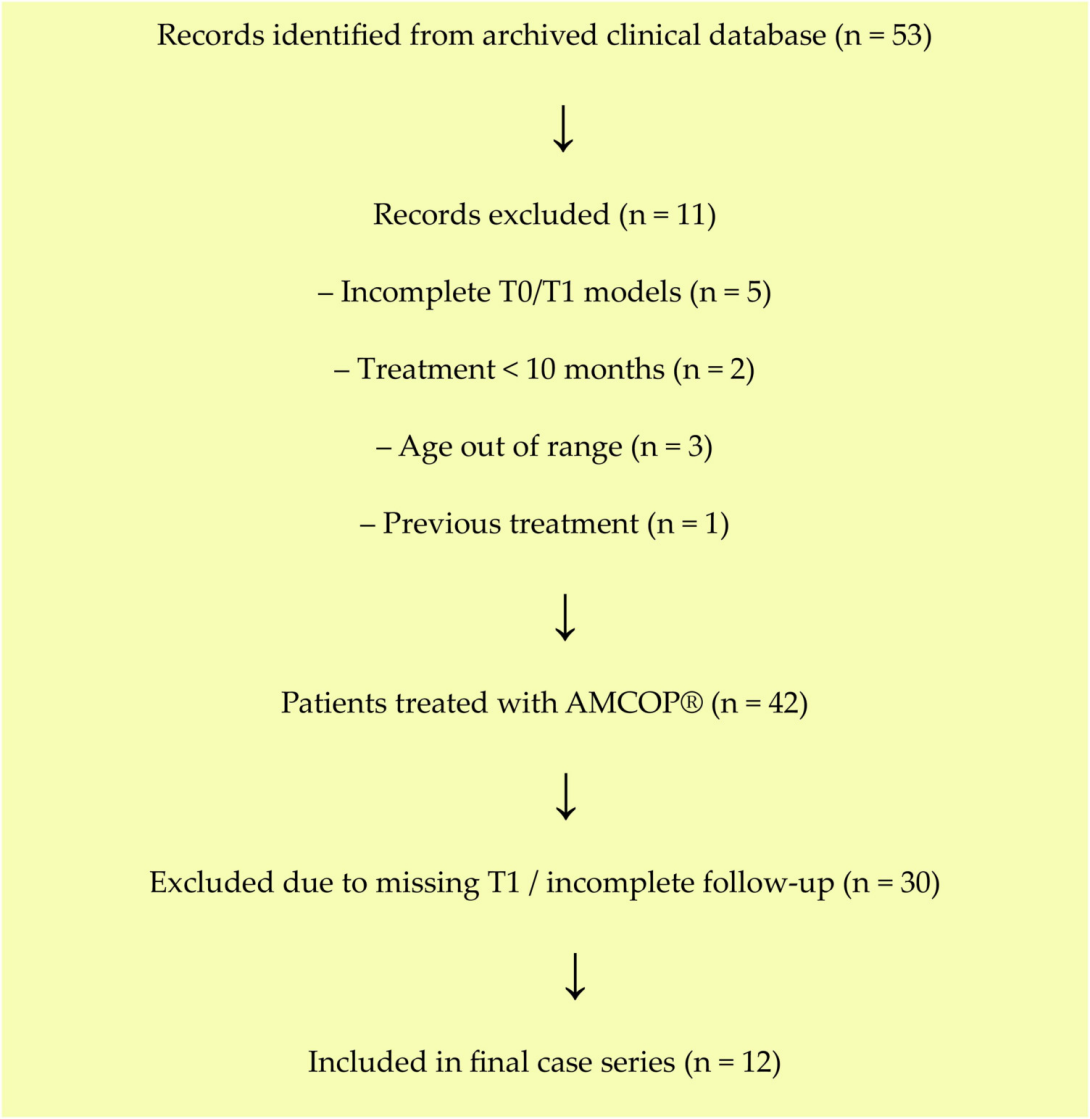
CONSORT-style flow diagram of patient selection. Flow diagram illustrating retrospective identification of growing patients treated with AMCOP® elastodontic appliances, exclusions with reasons, follow-up completeness, and inclusion in the final case series.

A total of *n* = 53 growing patients were assessed for eligibility from the archived clinical records. Of these, *n* = 11 were excluded (reasons: incomplete T0/T1 digital models *n* = 5; insufficient treatment duration <10 months *n* = 2; age out of range *n* = 3; previous transverse orthopedic treatment *n* = 1). The remaining *n* = 42 patients had been treated with AMCOP® appliances during the study period. Among these, *n* = 30 were excluded from the analysis due to missing post-treatment records or incomplete follow-up documentation. The final case series therefore included *n* = 12 patients (8 females, 4 males; mean age: 5.6 ± 0.8 years) with complete and analyzable paired T0–T1 digital models (and Teethan® sEMG recordings, where applicable) (Figure 1).

### 2.5 Functional orthopedic devices (AMCOP® system)

All patients were treated using AMCOP® (Apparecchi Modulari di Contenzione Ortopedica Personalizzati) elastodontic appliances [Micerium S.p.A. Registered office: Viale Beatrice D'Este 20, 20122 Milan (MI), Italy.

Operational and administrative headquarters: Via Guglielmo Marconi 83, 16036 Avegno (GE), Italy. Ortho Protec S.r.l., Bari, Italy], following the department's standardized functional orthopedic protocol.

Two main device categories were employed according to clinical needs and palatal morphology:

1. AMCOP® Integral—used as a first-phase appliance to release transverse compression, restore correct tongue posture, and achieve neuromuscular balance.
2. AMCOP® Class Devices (e.g., Class III)—used, when indicated, only after transverse normalization to refine sagittal relationships.

All devices were removable and worn daily according to the prescribed schedule (daytime and nocturnal use). Compliance was assessed through structured verbal parental reporting and chairside questioning during each scheduled 4–6-week follow-up visit. No objective wear-time sensors or electronic monitoring systems were used; therefore, adherence assessment relied solely on subjective reporting. Functional instructions, including nasal breathing, tongue posture and masticatory exercises, were reinforced at each 4–6-week follow-up visit. AMCOP® appliances were selected according to the patient's malocclusion and arch morphology. Device selection followed a standardized clinical protocol based on occlusal classification, palatal morphology, and transverse deficit distribution. Integral appliances were chosen in patients with Class I occlusion or transverse deficiency without sagittal discrepancy, while Class III (TC) devices were selected in the presence of anterior crossbite or skeletal Class III tendency requiring sagittal guidance. Arch form selection (S, OS, F, C) was based on visual and metric assessment of palatal shape (triangular, oval, square/oval, or circular) obtained from intraoral examination and digital models. Device size was determined directly using the manufacturer fitting protocol based on maxillary intermolar width and age-related size charts, aiming for gentle passive insertion without mucosal compression. For Class I malocclusions, the Integral line was used, available in different arch forms (S, OS, F, C); in the present sample, only the S and OS variants were employed. For Class III malocclusions, the TC device was used, which features a single arch form and a specific design of the occlusal plane that promotes maxillary advancement and provides mandibular anchorage. Although all devices share the same elastodontic principles, their morpho-functional differences must be considered when interpreting the clinical and neuromuscular outcomes.

After completion of active treatment, no retention appliances were prescribed, and no standardized post-treatment follow-up visits were scheduled. Therefore, outcome assessment was limited to the active treatment period only.

### 2.6 Device characteristics

The AMCOP® appliances used in this study are made from a polymer–elastomer blend available in two Shore hardness grades (51 and 60), selected according to functional requirements. The material is elastic and thermoplastic, showing heat-activated adaptability that allows the appliance to conform to individual arch forms. When immersed in hot water at approximately 70 °C for 30 s and then cooled in cold water, the device can undergo controlled expansion and shape stabilization, facilitating individualized fitting.

The AMCOP® bioactivators are available in various sizes, shapes, and colors, corresponding to different skeletal classes and dental arch morphologies. For this study, only devices designed for mixed dentition were used, primarily:

1. Class I Integral devices, indicated for basal skeletal discrepancies in the transverse and vertical planes.
2. Class III (TC) devices used when transverse contraction was associated with an anterior crossbite tendency.

**Figure 2:**
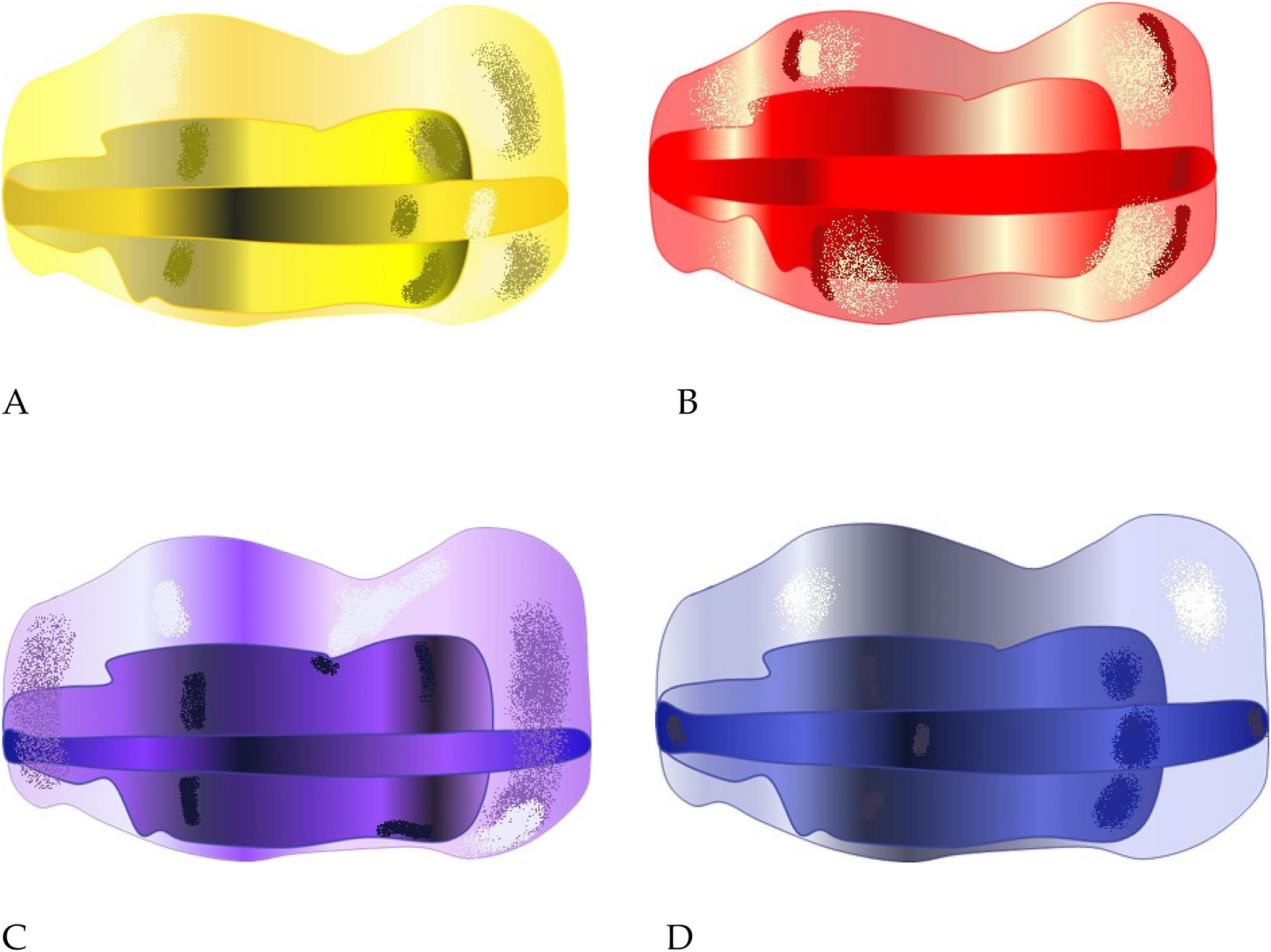
AMCOP® class I integral appliances, color-coded for morpho-functional variants: **(A)** F—triangular, **(B)** S—harmonic, **(C)** OS—oval-square, **(D)** C—circular. Variants differ in buccal contour, transverse width, and flange curvature. Each device differs in transverse width, flange curvature and palatal contour, allowing individualized fitting according to the patient's craniofacial morphology and transverse deficiency pattern.

Class I appliances are manufactured with four arch forms and two occlusal plane configurations (Figure 2):

- Arch shapes: F (triangular, dolichocephalic, narrow palate); S (harmonic, mesocephalic, oval palate); OS (oval-square, mesocephalic, broad arch); C (circular, brachycephalic, wide/flat palate).
- Occlusal planes: Integral (flat plane, normal bite) and Basic (thicker anterior plane, increased vertical dimension for deep bites).

In patients with anterior crossbite or sagittal discrepancies associated with transverse contraction, the AMCOP® Class III (TC) device was used. This elastodontic bioactivator exerts combined sagittal and transverse functional effects through its inclined occlusal planes and elastic flanges, promoting posterior mandibular repositioning, gradual transverse expansion, and neuromuscular reprogramming.

**Figure 3:**
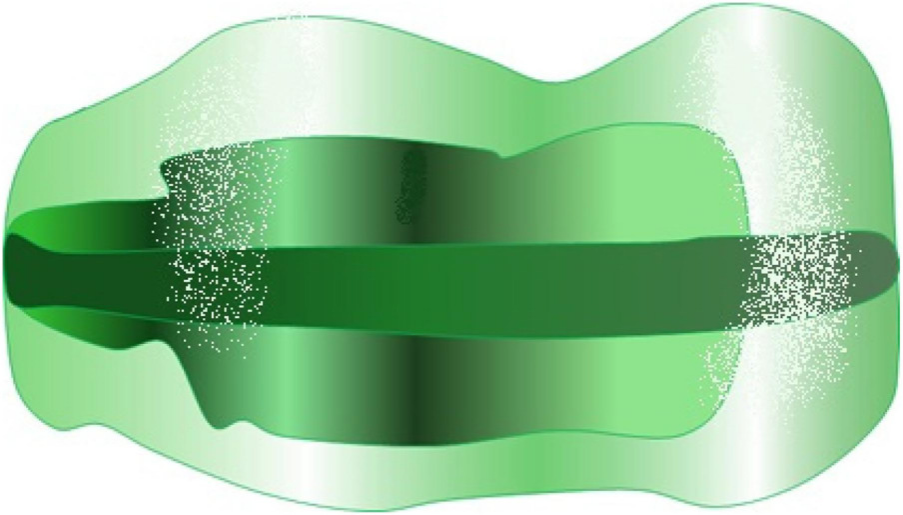
AMCOP® class III (TC) elastodontic appliance. The device features inclined occlusal planes for mandibular repositioning and elastic flanges providing gentle transverse expansion and neuromuscular reprogramming, specifically indicated for early Class III correction associated with maxillary transverse constriction.

The Class III device is made of a heat-activated polymer–elastomer blend (Shore 51/60) and features a dual-arch design with inclined occlusal planes guiding mandibular closure. Its flexible flanges promote gradual transverse expansion and neuromuscular rebalancing by centering the tongue and restoring a functional occlusion (Figure 3).

Selection of AMCOP® device variants followed a predefined clinical decision protocol based on sagittal occlusal class, presence of anterior crossbite, and transverse deficiency pattern. Integral appliances were selected for patients with Class I occlusion or isolated transverse maxillary deficiency without sagittal discrepancy. Class III (TC) devices were selected for patients presenting anterior crossbite or skeletal Class III tendency requiring sagittal guidance in addition to transverse correction.

Device size selection was based on manufacturer fitting guidelines using maxillary intermolar width measurements obtained from digital models and age-related size charts. The selected appliance size aimed to ensure gentle passive insertion without mucosal compression or excessive deformation. Arch form (S, OS, F, C) was chosen based on visual and metric assessment of palatal morphology derived from digital models (triangular, oval, square/oval, circular) (Table 1).

**Table 1:** Device selection criteria.

Clinical Feature	Integral Appliance	Class III (TC) Appliance
Sagittal occlusion class	Class I or no sagittal discrepancy	Skeletal Class III pattern
Anterior crossbite	Absent	Present
Primary treatment goal	Transverse development and functional reeducation	Transverse development + sagittal guidance
Appliance activation	Passive elastodontic function	Passive elastodontic function with sagittal guidance design
Size selection	Based on intermolar width and age charts	Based on intermolar width and age charts
Arch form selection	S, OS, F, C depending on palatal morphology	Single standardized arch form

### 2.7 Digital model acquisition and measurement protocol (Deltadent®)

Digital dental models were obtained using a 3Shape TRIOS 4 intraoral scanner (3Shape, Copenhagen, Denmark), with a declared accuracy of approximately 20–25 µm according to manufacturer specifications. Scanner calibration was performed following the standard manufacturer protocol prior to each scanning session. Digital impressions were obtained at baseline (T0) and at the end of transverse treatment (T1). Linear transverse measurements were performed on 3D models using Deltadent® software [Manufactured by Outside Format, Via Circonvallazione D 28, 26025 Pandino (CR), Italy].

Reference points and measurements:

1. Intercanine width: distance between the palatal gingival margins of upper canines (teeth 13 and 23).
2. Inter–second deciduous molar width (“inter-5”): distance between palatal cervical margins of teeth 55 and 65.
3. Intermolar width: distance between palatal gingival margins of first permanent molars (16 and 26), when erupted. Intermolar measurements were performed only in patients with fully erupted first permanent molars; no adjustment or imputation was applied for cases without inter-6 data.
4. Reference axis: mid-palatal raphe.

**Figure 4:**
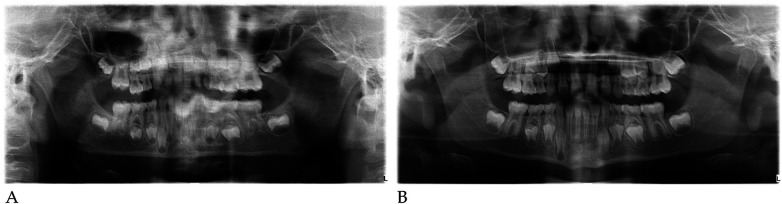
Pre- **(A)** and post-treatment **(B)** panoramic radiographs of case 1.

All measurements were taken by a single calibrated examiner. The examiner was not blinded to treatment stage (T0 vs. T1), due to the retrospective design and the evident chronological features of the digital models. A random subsample (20%) was remeasured after 7 days to assess intra-examiner reliability using the intraclass correlation coefficient (ICC) and Dahlberg's error. Intra-examiner reliability was excellent, with ICC values ranging from 0.92 to 0.98. Dahlberg's error ranged from 0.18 to 0.32 mm. No measurements exceeded the 0.5 mm threshold. Discrepancies > 0.5 mm were re-evaluated, and the average of the two closest values was recorded. The mid-palatal raphe was defined on the 3D models as the sagittal reference line passing through the median palatal suture, visually identified from the posterior nasal spine region to the incisive papilla and digitally traced within the Deltadent® software environment. The measurement plane was oriented perpendicular to this mid-sagittal axis. Transverse distances were recorded between standardized anatomical landmarks identified at the most cervical palatal gingival margin of each reference tooth (canines, second deciduous molars and, when erupted, first permanent molars). Landmark placement was performed in the axial palatal view with the model oriented with the occlusal plane parallel to the horizontal reference plane. Examples of landmark definition and placement on 3D models are shown in Figures 4, 8, 12, and subsequent case figures.

### 2.8 Neuromuscular assessment (Teethan® surface electromyography)

Neuromuscular balance was assessed through surface electromyography (sEMG) using a portable Teethan® system [Teethan S.p.A. Headquarters: Viale Forlanini 42/A, 20024 Garbagnate Milanese (MI), Italy]. Recordings were obtained at T0 and T1 under standardized conditions (maximum intercuspation in habitual occlusion). Electrode type and placement followed the manufacturer's standardized protocol and SENIAM guidelines for facial sEMG. Disposable Ag/AgCl surface electrodes (diameter 24 mm) were used. After skin cleaning with alcohol, bipolar electrodes were positioned bilaterally over the muscle bellies of the anterior temporalis (TA) and masseter (MM) muscles, with an interelectrode distance of approximately 20 mm, parallel to the muscle fibers. Reference electrodes were positioned according to the system protocol. For postural assessment, electrodes were similarly placed over the sternocleidomastoid (SCM) muscles.

Signals were acquired at a sampling rate of 1,000 Hz and processed using the integrated Teethan® software algorithms, including band-pass filtering and artifact rejection according to the manufacturer's validated protocol. Recordings were obtained during maximal voluntary clenching in habitual intercuspation for 5 s, and three consecutive trials were performed. The trial showing the greatest signal stability was selected for analysis. Data were internally normalized by the Teethan® system using relative bilateral muscle activity ratios, thus no external MVC normalization was required.

All examinations were conducted by the same trained operator following standardized instructions. Although blinding to treatment timing was not feasible due to the clinical design, all recordings were performed under identical acquisition settings to ensure test–retest consistency. The “Impact index” (IMP) provided by the Teethan® system is a normalized, dimensionless score reflecting global muscular efficiency and recruitment quality. According to the manufacturer's validation protocol, IMP is scaled so that a value around 100% corresponds to age- and population-referenced normative mean efficiency; values substantially below 100% indicate reduced muscular recruitment, whereas values above 100% indicate muscular hyperactivity or incoordination beyond normative baseline. Therefore, an increase of 20–30 percentage points in IMP, or a shift from values well below 50% to ≈100%–120%, as observed in most treated cases, can be interpreted as a clinically meaningful improvement in neuromuscular efficiency, consistent with previously published normative data (Rocchetti et al., 2009; Tartaglia et al., 2011).

The following muscles were monitored: anterior temporalis (TA), masseter (MM), and sternocleidomastoid (SCM).

Functional indices analyzed included:

- POC TA/MM (%): right/left symmetry of activation.
- BAR (Barycenter): anterior–posterior functional balance.
- TORS: functional torsion (right/left dominance).
- ASIM: overall asymmetry index.
- POC SCM (%): cervical postural symmetry.
- IMP/CL: global intensity and co-contraction level.

Each electromyographic index reflects a specific aspect of neuromuscular balance:

- POC TA/MM expresses bilateral activation symmetry of the anterior temporalis and masseter muscles (%);
- BAR (Barycenter) indicates the antero-posterior distribution of muscular activity and functional mandibular positioning;
- TORS describes right–left dominance and mandibular torsional imbalance;
- ASIM represents an overall asymmetry coefficient between muscle pairs;
- IMP (Impact Index) reflects global muscular efficiency and strength of recruitment;
- CL (Co-contraction Level) represents the degree of muscle coactivation during maximal intercuspation.

Values within the physiological range indicate balanced neuromuscular function. Normative thresholds for each index were automatically provided by the validated Teethan® database and were applied consistently across all measurements.

The sEMG data were correlated with morphometric changes (ΔT1–T0) to evaluate neuromuscular rebalancing following transverse correction. A summary of all sEMG functional indices, their definitions, and clinical significance is provided in Table 2.

**Table 2:** Description of sEMG functional indices recorded with the Teethan® system.

Abbreviation	Full name	Functional meaning
POC TA/MM	Percentage of Overlapping Coefficient	Bilateral symmetry of anterior temporalis and masseter muscle activation (%)
BAR	Barycenter	Antero-posterior distribution of muscular activity and functional mandibular position
TORS	Torsion index	Right–left dominance and mandibular torsional imbalance
ASIM	Asymmetry index	Global inter-muscular asymmetry measurement
IMP	Impact Index	Muscular recruitment efficiency and contraction strength
CL	Co-contraction Level	Degree of synchronized muscle activation during maximal intercuspation
POC SCM	Percentage of Overlapping Coefficient SCM	Bilateral symmetry of sternocleidomastoid muscle activation (%)

### 2.9 Normative thresholds and interpretation of teethan® indices

Interpretation of sEMG indices was based on normative reference values provided by previous validation studies of the Teethan® system [Rocchetti 2009; Tartaglia 2011; Ferrario & Sforza 2012].

The following cut-off values were applied:

- POC TA/MM and POC SCM: values ≥85% indicate physiological bilateral symmetry; values <85% indicate muscular asymmetry.
- BAR (Barycenter): optimal values between 85%–115% indicate balanced antero–posterior distribution of muscular activity; values <85% or >115% indicate posterior or anterior imbalance.
- TORS: physiological range between 85%–115%; values outside this interval indicate mandibular torsional imbalance.
- ASIM: physiological values ≤10%; higher absolute values indicate global muscular asymmetry.
- IMP: optimal efficiency values between 85%–115%; values <85% indicate hypo-activation, while values >115% indicate hyper-activation.
- CL (Co-contraction Level): physiological values ≤15%, reflecting normal muscular synchronization during maximal intercuspation.

Accordingly, the terms “normalized” and “within limits” used throughout the manuscript refer to indices shifting into these validated physiological intervals at T1.

### 2.10. Outcomes

Primary outcomes:

- Changes (T1–T0) in intercanine, inter-5, and intermolar widths (mm).

Secondary outcomes:

- Improvement of neuromuscular indices (POC, BAR, TORS, ASIM, POC-SCM, IMP/CL).
- Clinical correction of posterior crossbite or transverse arch constriction.

### 2.11. Sample size and study power

This study was designed as an observational case series of 12 consecutively treated patients, aimed at documenting the clinical and functional effects of AMCOP® therapy.

This study was designed as an exploratory observational case series of 12 consecutively treated patients. No *a priori* sample size calculation was performed, as the primary aim was descriptive: to document morphometric and neuromuscular trends associated with AMCOP® therapy in early mixed dentition.

Considering the absence of inferential analysis, *post-hoc* evaluation focused on the magnitude of observed changes (effect size in a clinical sense). Mean transverse gains ranging from approximately 3 to 5 mm represent clinically meaningful effects for interceptive orthopedic therapy in this age group.

### 2.12. Data management and quality control

All data were pseudonymized and securely stored in the department's protected database.

Digital models, sEMG recordings, and clinical photographs were analyzed within a standardized workflow.

Periodic quality checks verified the consistency of landmark identification and the reproducibility of measurements.

### 2.13. Statistical considerations

Given the retrospective case-series design and the limited sample size, no inferential statistical analyses were planned or performed. No hypothesis testing, confidence intervals, or significance thresholds were applied. Data were summarized descriptively as mean ± standard deviation and range to describe within-subject pre–post variations. In addition, *post-hoc* standardized mean change (SMC) effect sizes were calculated for the primary transverse outcomes using the ratio of mean change to standard deviation of the change scores. Effect size values were interpreted according to Cohen's thresholds (0.2 small, 0.5 moderate, 0.8 large). Results should therefore be considered exploratory and descriptive rather than evidence of treatment efficacy.

## 3 Case series

### 3.1 Case 1—C.S., female, 5 years

Dentition stage: Early mixed dentition.

Diagnosis: Maxillary transverse deficiency with mild unilateral posterior crossbite.

Appliance: AMCOP® Integral S; worn 1 h per day plus every night, for a total of 12 months.

#### 3.1.1 Radiographic assessment

Pre- and post-treatment panoramic radiographs confirmed normal root development and eruption pattern.

The post-treatment image showed adequate space for the eruption of the permanent incisors and early molars, without dental or skeletal asymmetries (Figures 4A,B).

#### 3.1.2 Digital model analysis

**Figure 5:**
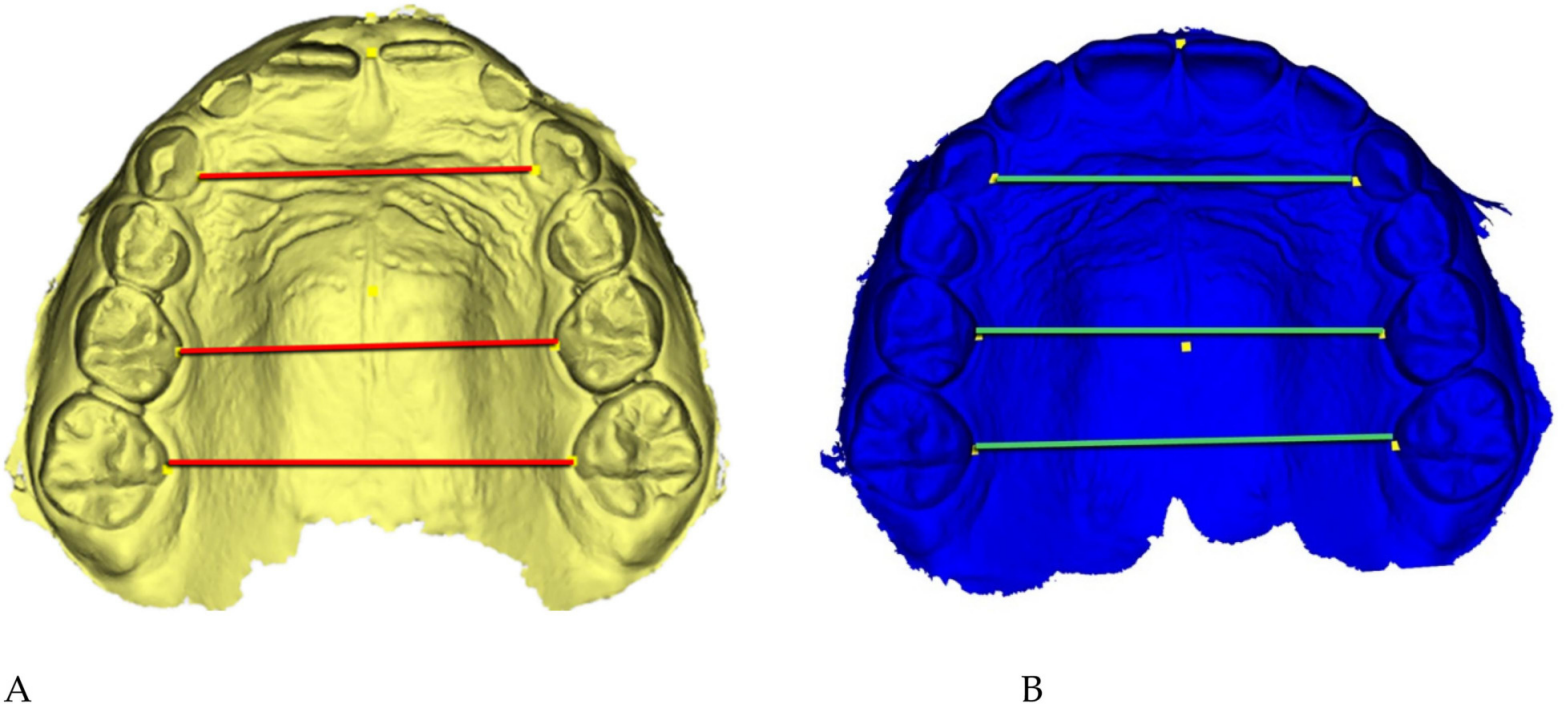
Digital 3D models: **(A)** pre-treatment (yellow), **(B)** post-treatment (blue). Colored guidelines correspond to the measured inter-dental distances and are shown for visual reference only.

Digital 3D models obtained with *Deltadent®* were analyzed for transverse measurements (Figures 5A,B).

The reference landmarks corresponded to the palatal gingival margins of the upper canines, second deciduous molars, and first permanent molars when erupted.

Transverse measurements (Table 3) were associated with increases observed in both the anterior and posterior maxillary regions.

**Table 3:** Transverse measurements on digital models (yellow = pre-treatment; blue = post-treatment). Δ = absolute change (post−pre); Δ% = percent change relative to pre-treatment value.

Distance	Pre (mm)	Post (mm)	Δ (mm)	Δ %
Intercanine	27.24	30.98	+3.74	+13.7%
Inter-5 (second deciduous molars)	30.60	34.60	+4.00	+13.1%
Inter-6 (first molars)	33.03	36.11	+3.08	+9.3%

Qualitative 3D inspection confirmed a symmetrical transverse expansion, improved occlusal interdigitation, and enhanced arch coordination (Figure 4).

#### 3.1.3 Digital bite evaluation

**Figure 6:**
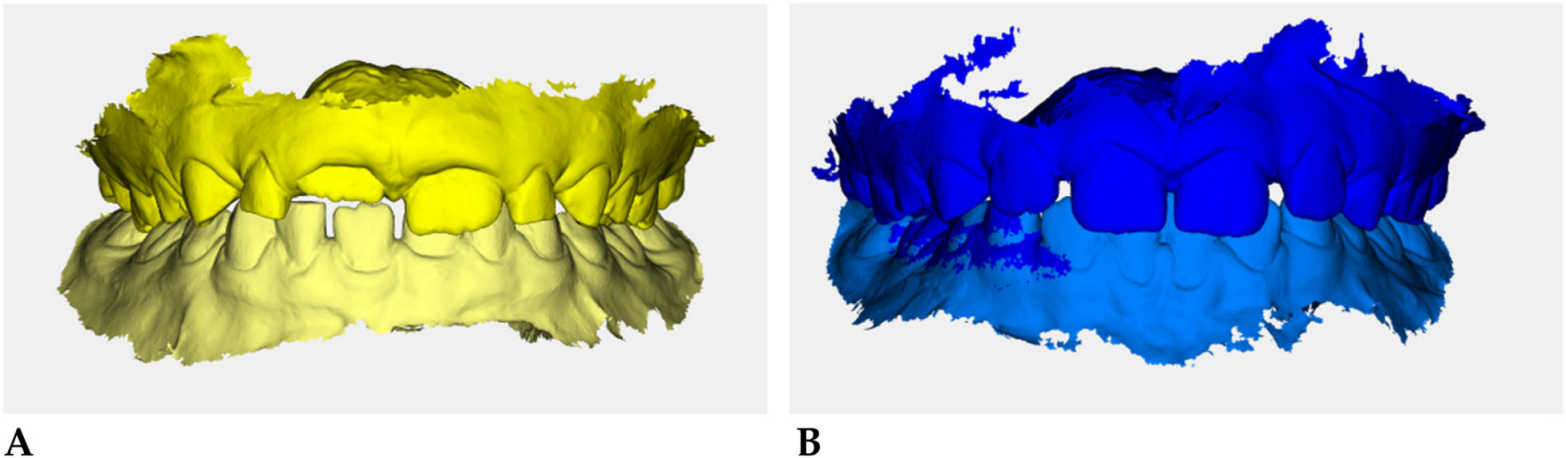
Digital occlusal models at pre- **(A)** and post-treatment **(B)** Pre-treatment models **(A)** are color-coded in yellow, with two shades (darker = maxillary, lighter = mandibular) to distinguish the arches; post-treatment models **(B)** are color-coded in blue. These renders provide a qualitative overview of transverse coordination and occlusal improvement obtained with elastodontic therapy.

Color-coded occlusal digital models were generated for qualitative comparison of the maxillary and mandibular arches (Figures 6A,B).

#### 3.1.4 Surface electromyography (Teethan®) assessment

Neuromuscular activity was recorded at baseline and after 12 months of treatment using *Teethan®* sEMG.

**Figure 7:**
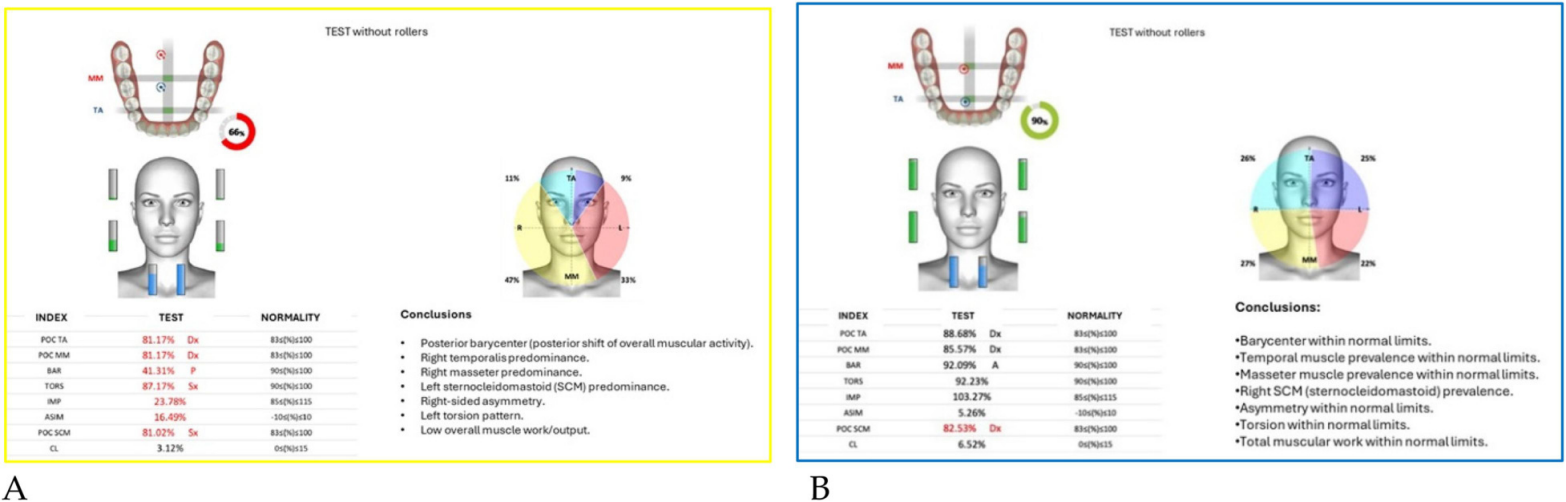
Teethan® surface electromyography [**(A)**: pre-treatment; **(B)**: post-treatment]. Post-treatment data show restoration of muscle symmetry and improved functional barycenter distribution.

The analysis revealed marked improvement in muscle symmetry and coordination, particularly in the temporalis (TA) and masseter (MM) pairs (Table 4, Figure 7).

**Table 4:** sEMG functional indices before and after AMCOP® therapy.

Index	Pre	Post	Outcome
POC TA	81.17%	88.68%	From below → within range
POC MM	81.17%	85.57%	Improved
BAR (Barycenter)	41.31%	92.09%	Normalized
TORS	87.17% (L)	92.23%	Normalized
IMP	23.78%	103.27%	Normalized
ASIM	16.49%	5.26%	Reduced to normal range
POC SCM	81.02%	82.53%	Slight improvement
CL	3.12%	6.52%	Within range

#### 3.1.5 Interpretation

After 12 months of AMCOP® Integral S therapy, the patient showed:

- Clinically significant transverse widening (+3.1 to +4.0 mm at all reference points);
- Balanced arch form with improved occlusal coordination;
- Neuromuscular normalization on sEMG, including restored barycenter position and reduced asymmetry indices.

Minor residual variability in SCM activation (≈82.5%) was interpreted as within physiological limits for a 5-year-old, likely influenced by cooperation during EMG recording rather than by true dysfunction.

Overall, the observed combination of transverse dimensional changes and neuromuscular index variations was associated with early elastodontic intervention.

### 3.2 Case 2—V.S., male, 7 years

Dentition stage: Early mixed dentition.

Diagnosis: Maxillary transverse deficiency associated with a mild skeletal Class III pattern.

Appliance: AMCOP® Class III (TC); worn 1 h per day plus every night, for a total of 10 months.

#### 3.2.1 Radiographic assessment

**Figure 8:**
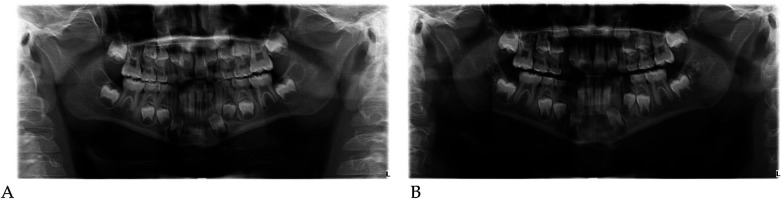
Pre- **(A)** and post-treatment **(B)** panoramic radiographs of case 2.

Panoramic radiographs (Figure 8) were obtained to document dental development and eruption sequence.

No skeletal anomalies or asymmetric root morphology were noted, confirming suitability for functional elastodontic therapy.

#### 3.2.2 Digital model analysis (palatal view)

Digital 3D models obtained with *Deltadent®* were analyzed at baseline (T0) and after 10 months (T1).

Quantitative measurements (Table 5) show homogeneous anterior and posterior expansion, with a gain of approximately **+**4.6 mm intercanine and +4.9 mm intermolar, indicating a balanced and effective transverse correction.

**Table 5:** Transverse measurements on digital models (yellow = pre-treatment; blue = post-treatment). Δ = absolute change (post−pre); Δ% = percent change relative to pre-treatment value.

Distance	Pre (mm)	Post (mm)	*Δ* (mm)	Δ %
Intercanine (C–C)	22.45	27.04	+4.59	+20.5%
Inter–second deciduous molars (5–5)	29.14	34.02	+4.88	+16.7%
Inter–first molars (6–6)	30.55	35.42	+4.87	+15.9%

#### 3.2.3 Clinical interpretation

The patient exhibited a harmonic transverse expansion, with comparable anterior and posterior widening.

**Figure 9:**
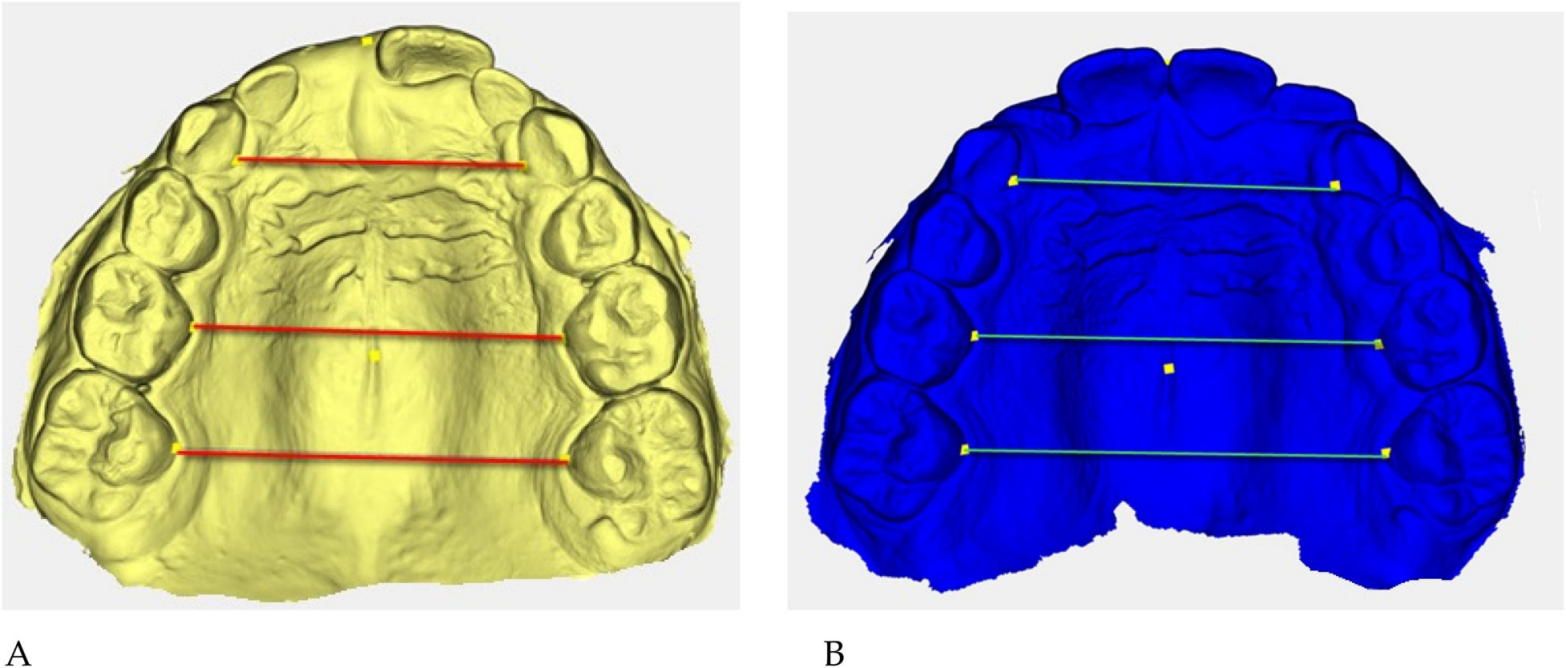
Digital 3D models of case 2 [**(A)**: pre-treatment; **(B)**: post-treatment]. Yellow = pre-treatment; blue = post-treatment. Colored guide lines correspond to the inter-dental distances measured with Deltadent®.

Given the patient's age (7 years) and the 10-month therapy duration with an AMCOP® Class III device, the increase is clinically significant; a small portion may reflect normal growth, but the magnitude clearly suggests a therapeutic effect (Figure 9).

#### 3.2.4 Digital bite evaluation

**Figure 10:**
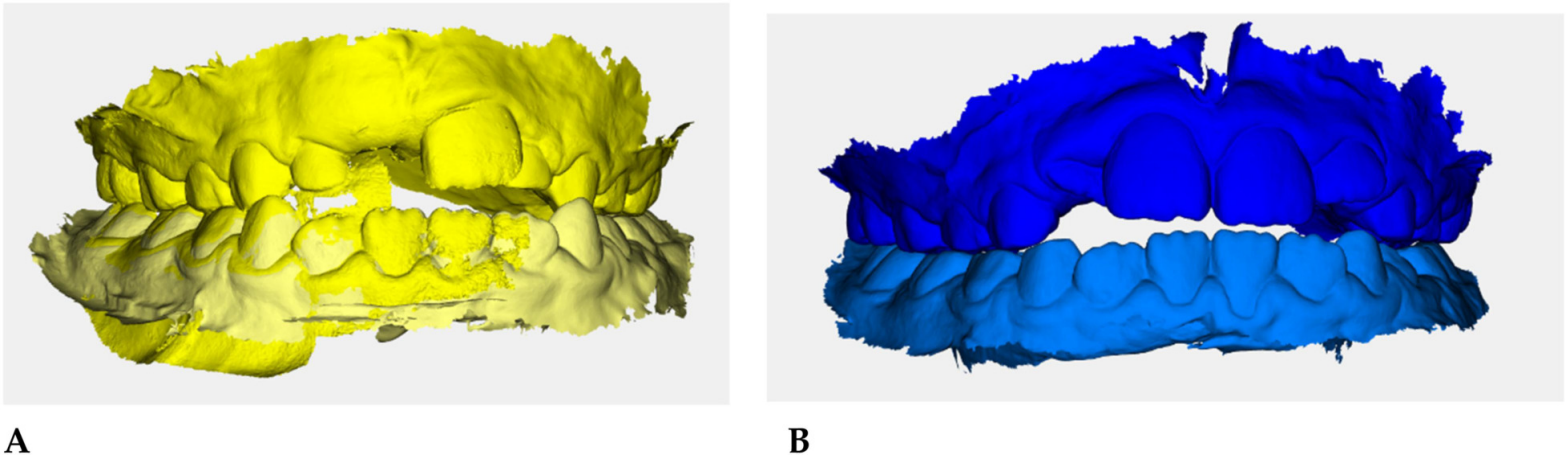
Digital occlusal models at pre- **(A)** and post-treatment **(B)** Pre-treatment model **(A)** = yellow (darker = maxillary, lighter = mandibular); post-treatment **(B)** = blue.

Color-coded occlusal digital models (Figures 10A,B) were used to qualitatively assess changes in occlusal coordination.

The post-treatment model shows improved arch form balance, correction of anterior crossbite tendency, and better interdigitation between upper and lower arches (Figure 10).

#### 3.2.5 Surface electromyography (Teethan®) assessment

Neuromuscular function was analyzed with *Teethan® sEMG* at baseline and after 10 months of treatment.

**Figure 11:**
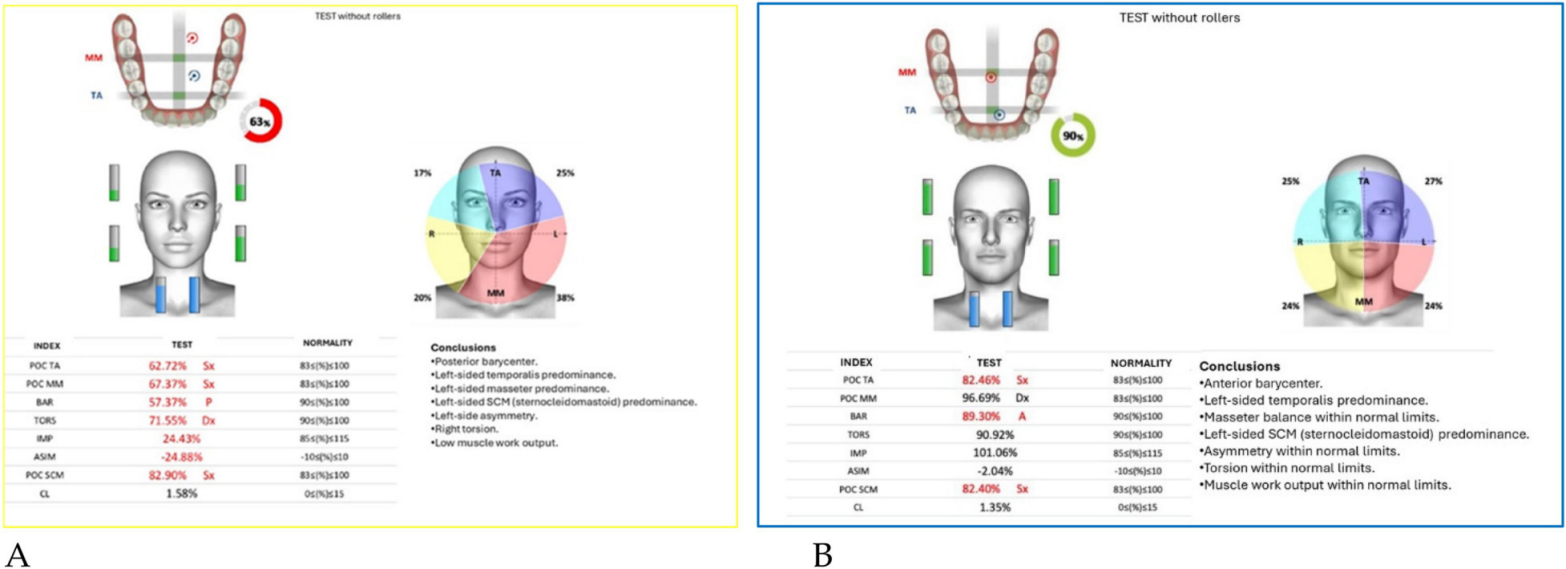
Teethan® surface electromyography [**(A)**: pre-treatment; **(B)**: post-treatment]. Post-treatment data show normalization of the barycenter, balanced temporalis and masseter activity and reduction of asymmetry and torsion indices.

Functional indices demonstrated a marked normalization of muscle coordination and barycenter alignment (Table 6, Figures 11A,B).

**Table 6:** sEMG functional indices before and after AMCOP® TC therapy.

Index	Pre	Post	Outcome
POC TA	85.41%	86.33%	Within normal limits
POC MM	56.81%	88.39%	From below → normalized
BAR	85.12%	90.62%	Normalized
TORS	79.57%	90.53%	Normalized
IMP	134.58%	88.59%	Reduced to normal limits
ASIM	28.89%	0.55%	Markedly reduced
POC SCM	83.32%	82.65%	Stable, within range
CL	6.67%	10.91%	Within normal limits

#### 3.2.6 Interpretation

After 10 months of AMCOP® TC therapy, the patient demonstrated:

- Significant and uniform transverse expansion (+4.5 to +4.9 mm at all levels);
- Resolution of anterior crossbite tendency and improved transverse coordination;
- Restoration of neuromuscular symmetry, with normalization of POC, BAR, and TORS values.

The marked improvement in interarch coordination and muscle balance supports the functional orthopedic efficacy of AMCOP® Class III elastodontic therapy in guiding early craniofacial growth.

### 3.3 Case 3—M.P., female, 6 years

Dentition stage: Early mixed dentition.

Diagnosis: Maxillary transverse deficiency with anterior constriction tendency, associated with mild skeletal Class III pattern.

Appliance: AMCOP® Integral, shape S; worn 1 h per day plus every night, for a total of 16 months.

#### 3.3.1 Radiographic assessment

**Figure 12:**
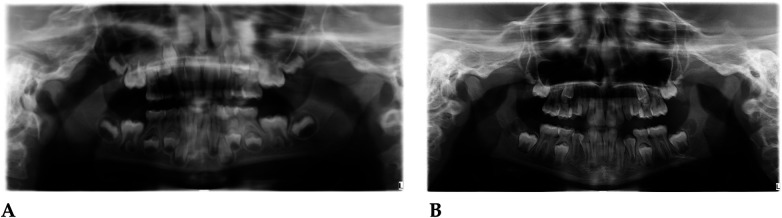
Pre- **(A)** and post-treatment **(B)** panoramic radiographs of case 3.

Panoramic radiographs (Figure 12) confirmed regular dental development and symmetric root morphology.

The eruption sequence was appropriate for the age, with adequate space for the eruption of permanent incisors and first molars.

No skeletal asymmetries were detected.

#### 3.3.2 Digital model analysis (palatal view)

Digital 3D models were obtained at baseline (T0) and after 16 months of therapy (T1) using *Deltadent®* software.

**Figure 13:**
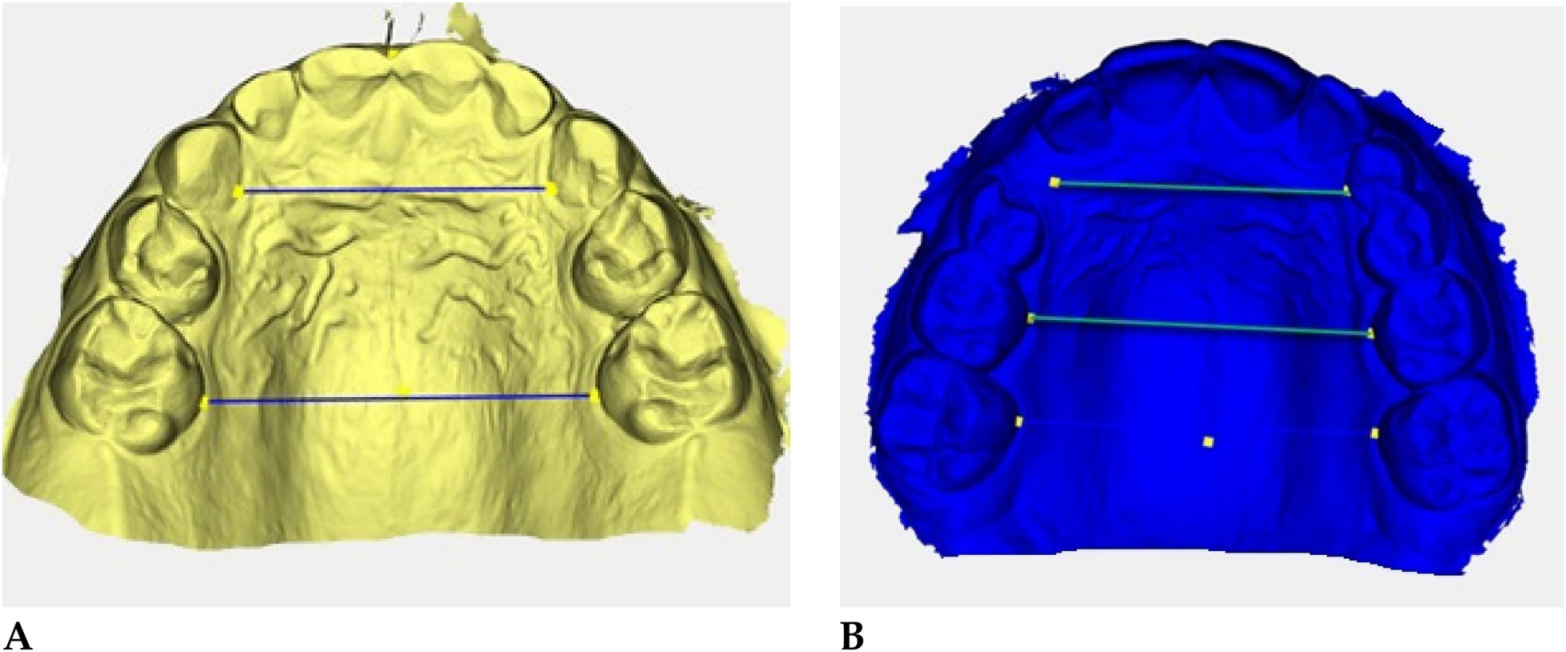
Digital 3D models of case 3 [**(A)**: pre-treatment; **(B)**: post-treatment]. Yellow = pre-treatment; blue = post-treatment. Colored guidelines correspond to the transverse distances analyzed.

Transverse measurements revealed a more pronounced anterior than posterior expansion, consistent with the expected action of the Integral device (Table 7, Figures 13A,B).

**Table 7:** Transverse measurements on digital models (yellow = pre-treatment; blue = post-treatment). Δ = absolute change (post−pre); Δ% = percent change relative to pre-treatment value.

Distance	Pre (mm)	Post (mm)	Δ (mm)	Δ %
Intercanine (C–C)	20.32	24.56	+4.24	+20.9%
Inter–second deciduous molars (5–5)	25.38	28.70	+3.32	+13.1%
Inter–first molars (6–6)	n.a.	29.95	—	—

#### 3.3.3 Clinical interpretation

The transverse expansion was greater in the anterior region (≈+4.2 mm) than posteriorly (≈+3.3 mm), in accordance with the selective anterior action of the Integral appliance.

The intercanine increase corresponds to an average of 0.42 mm/month, representing a clinically meaningful gain for widening the anterior corridor and improving the sagittal relationship typical of Class III cases.

#### 3.3.4 Digital bite evaluation

**Figure 14:**
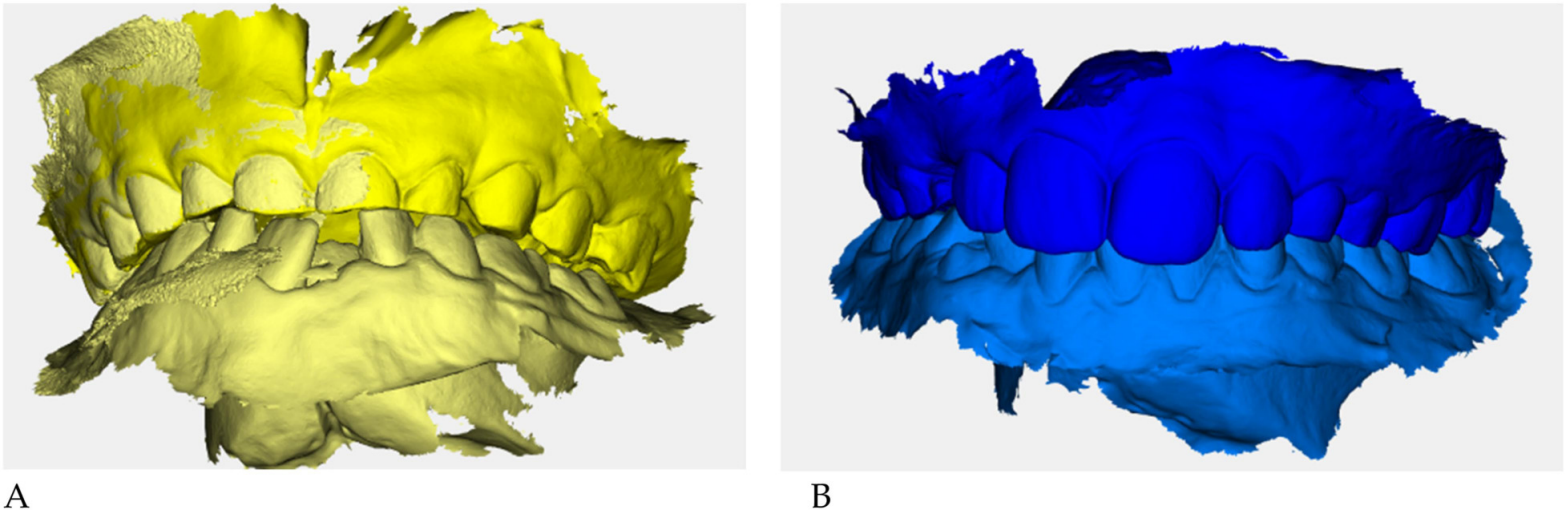
Digital occlusal models at pre- **(A)** and post-treatment **(B)**. Pre-treatment models **(A)** are color-coded in yellow (darker shade for maxillary, lighter for mandibular arches); post-treatment models **(B)** are color-coded in blue. The images provide a qualitative overview of transverse improvement and occlusal balance.

Color-coded occlusal models (Figures 14A,B) showed improved anterior arch coordination and increased transverse width.

Post-treatment images demonstrated enhanced occlusal interdigitation and symmetry between the maxillary and mandibular arches.

#### 3.3.5 Surface electromyography (Teethan®) assessment

Neuromuscular recordings using *Teethan® sEMG* were obtained before and after treatment.

**Figure 15:**
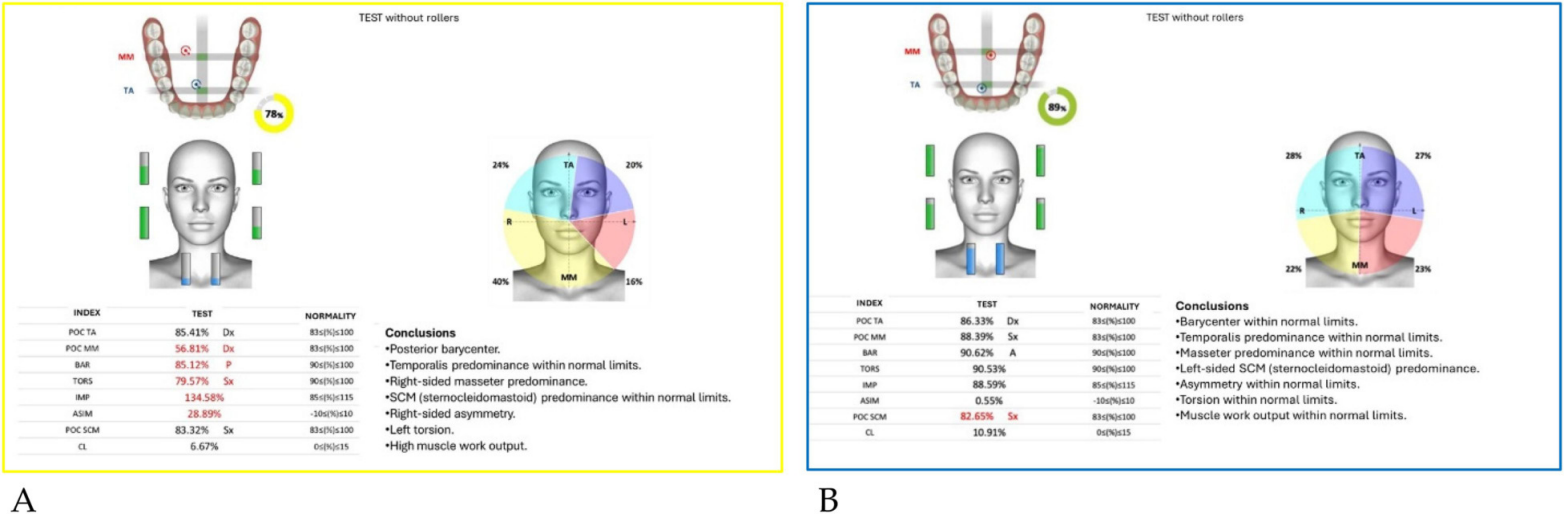
Teethan® surface electromyography [**(A)**: pre-treatment; **(B)**: post-treatment]. Post-treatment values show normalization of the barycenter, reduction of asymmetry and torsion, and balanced activation of temporalis and masseter muscles.

Functional indices indicated a clear improvement in muscle coordination, with normalization of barycenter and asymmetry parameters (Table 8, Figures 15A,B).

**Table 8:** sEMG functional indices before and after AMCOP® integral S therapy.

Index	Pre	Post	Outcome
POC TA	85.41%	86.33%	Within normal limits
POC MM	56.81%	88.39%	From below → normalized
BAR	85.12%	90.62%	Normalized
TORS	79.57%	90.53%	Normalized
IMP	134.58%	88.59%	Reduced to normal limits
ASIM	28.89%	0.55%	Markedly reduced
POC SCM	83.32%	82.65%	Stable, within range
CL	6.67%	10.91%	Within normal limits

#### 3.3.6 Interpretation

After 16 months of AMCOP® Integral S therapy, the patient showed:

- Greater anterior than posterior expansion, consistent with the appliance's selective effect;
- Transverse gain of 4.2 mm intercanine and 3.3 mm intermolar, with visible improvement in occlusal coordination;
- Normalization of neuromuscular indices, including balanced masseter and temporalis activation and corrected barycenter position.

The treatment resulted in a stable transverse expansion and functional rebalancing, confirming the efficiency of AMCOP® elastodontic therapy in early mixed dentition.

### 3.4 Case 4—I.R., female, 6 years

Dentition stage: Early mixed dentition.

Diagnosis: Maxillary transverse deficiency with constricted arch form and mild functional imbalance.

Appliance: AMCOP® Integral OS; worn 1 h per day plus every night, for a total of 12 months.

#### 3.4.1 Radiographic assessment

**Figure 16:**
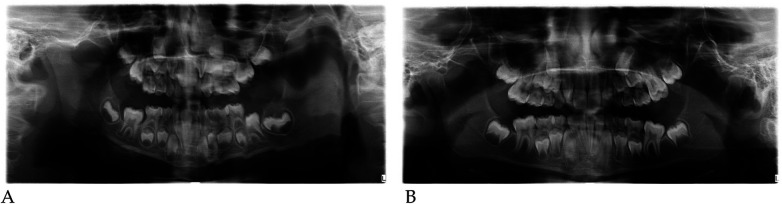
Pre- **(A)** and post-treatment **(B)** panoramic radiographs of case 4.

Panoramic radiographs (Figure 16) revealed normal root morphology and symmetrical eruption paths of both arches.

No skeletal asymmetries or pathologic findings were detected.

Adequate eruption space for permanent incisors and molars was observed after treatment.

#### 3.4.2 Digital model analysis (palatal view)

Digital 3D models were analyzed using *Deltadent®* software.

**Figure 17:**
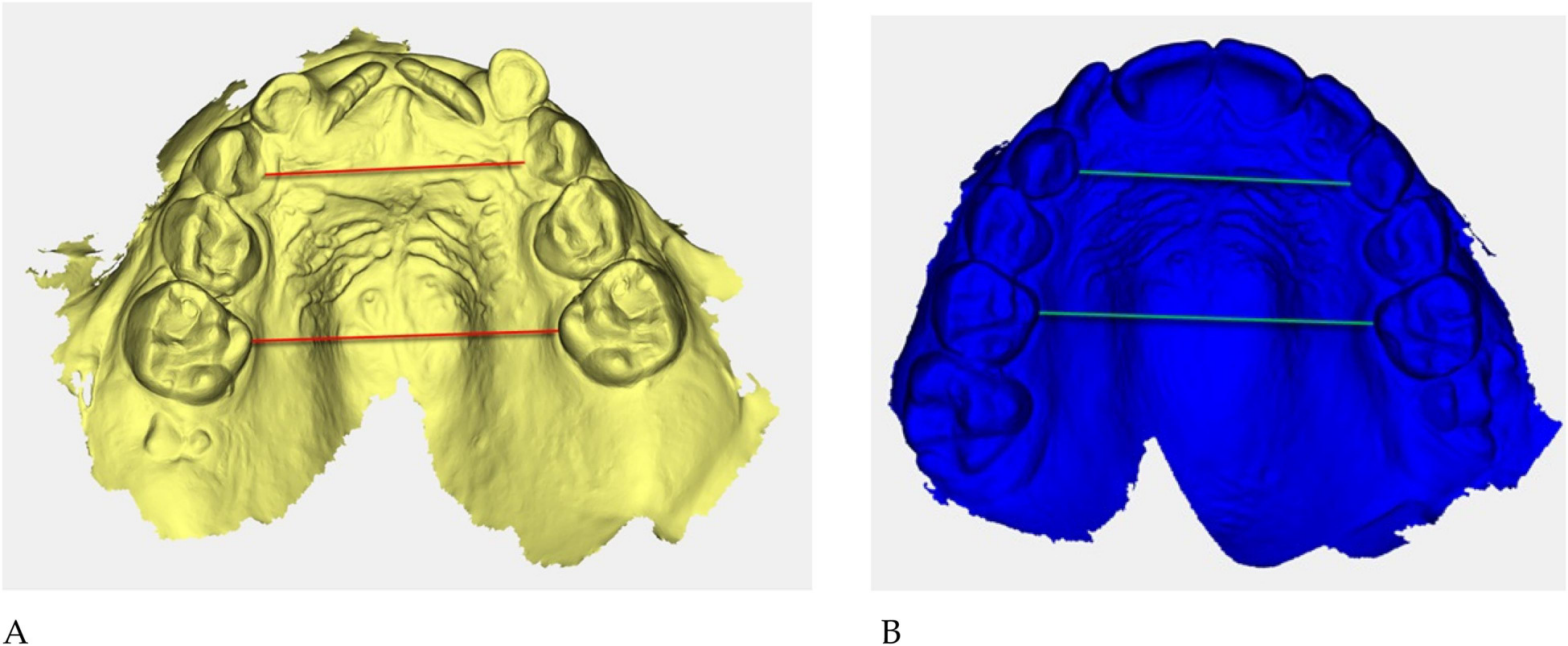
Digital 3D models of case 4 [**(A)**: pre-treatment; **(B)**: post-treatment]. Yellow = pre-treatment; blue = post-treatment. Colored reference lines correspond to the inter-dental distances measured in the analysis.

Quantitative data demonstrated a uniform and clinically significant expansion of the maxillary arch both anteriorly and posteriorly (Table 9, Figure 17).

**Table 9:** Transverse measurements on digital models (yellow = pre-treatment; blue = post-treatment). Δ = absolute change (post−pre); Δ% = percent change relative to pre-treatment.

Distance	Pre (mm)	Post (mm)	Δ (mm)	Δ %
Intercanine (C–C)	21.08	25.06	+3.98	+18.9%
Inter–second deciduous molars (5–5)	24.69	30.95	+6.26	+25.3%

#### 3.4.3 Clinical interpretation

After 12 months of AMCOP® Integral OS therapy, the patient showed marked skeletal widening, particularly in the posterior region (+6.3 mm), indicating a balanced orthopedic response and correction of the maxillary constriction.

The expansion pattern was harmonic, with well-maintained symmetry and improved palatal morphology.

#### 3.4.4 Digital bite evaluation

**Figure 18:**
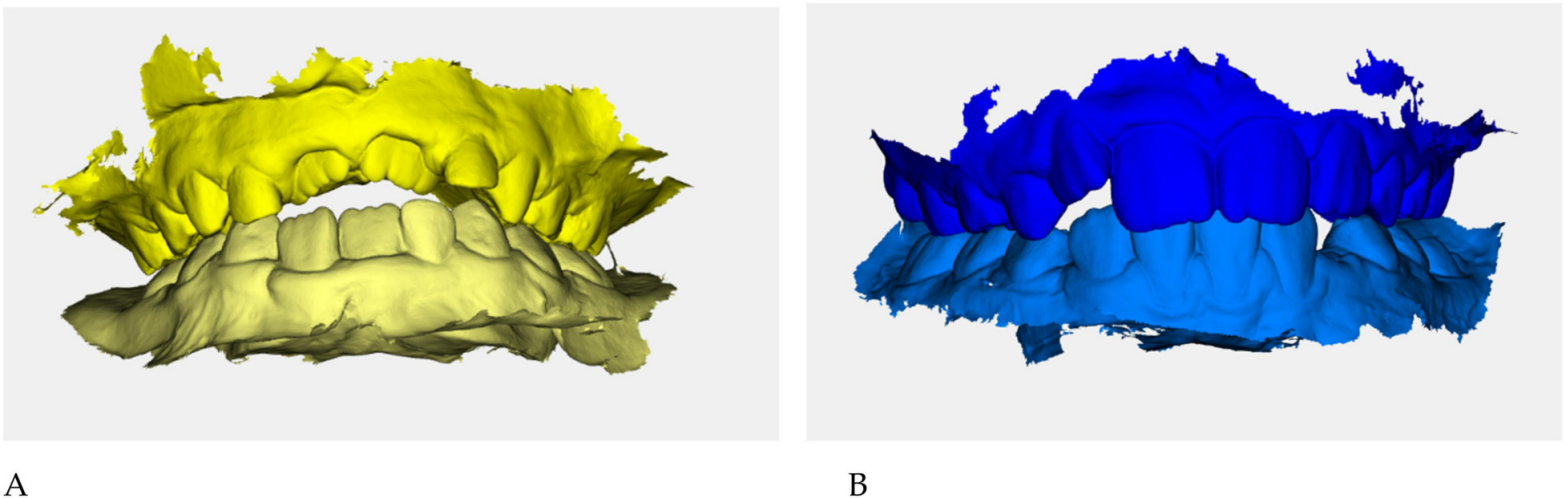
Digital occlusal models at pre- **(A)** and post-treatment **(B)**. Pre-treatment models **(A)** are color-coded in yellow (darker = maxillary, lighter = mandibular); post-treatment models **(B)** are in blue. Images illustrate improved transverse coordination and occlusal balance after therapy.

Color-coded occlusal digital models (Figures 18A,B) demonstrated improved arch coordination and occlusal interdigitation following treatment.

The post-treatment model (blue) shows balanced alignment between upper and lower arches and a wider anterior arch form.

#### 3.4.5 Surface Electromyography (Teethan®) assessment

Neuromuscular activity was recorded using *Teethan® sEMG* before and after AMCOP® therapy.

At baseline, electromyographic data revealed a posterior barycenter and mild right-side predominance.

**Figure 19:**
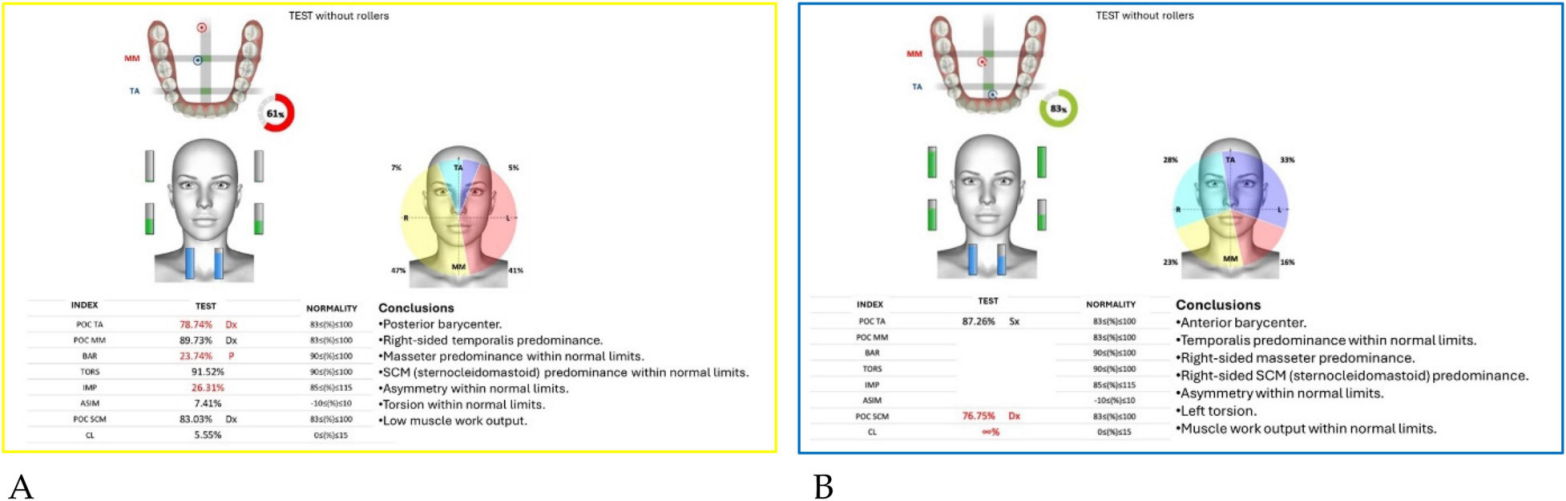
Teethan® surface electromyography [**(A)**: pre-treatment; **(B)**: post-treatment]. The post-treatment sEMG showed normalization of the barycenter, improved temporalis–masseter coordination, and reduction of asymmetry indices.

After treatment, all indices normalized, with barycenter repositioning and muscle symmetry restoration (Table 10, Figures 19A,B).

**Table 10:** sEMG functional indices before and after AMCOP® integral OS therapy.

Index	Pre	Post	Outcome
POC TA	78.74%	87.26%	From below → normalized
POC MM	89.73%	83.00%	Within normal limits
BAR	23.74%	90.00%	Normalized (posterior → anterior)
TORS	91.52%	90.50%	Stable, within normal limits
IMP	26.31%	85.00%	Normalized
ASIM	7.41%	0.00%	Symmetry restored
POC SCM	83.03%	76.75%	Mild left shift, within tolerance
CL	5.55%	10.00%	Within normal range

#### 3.4.6 Interpretation

After 12 months of AMCOP® Integral OS therapy, the patient exhibited:

- Significant transverse expansion, particularly posteriorly (+6.3 mm);
- Balanced arch development and restoration of occlusal coordination;
- Normalization of neuromuscular activity, with barycenter repositioned from posterior to anterior and reduced torsional asymmetry.

The combined morphometric and sEMG data confirm the efficacy of elastodontic functional therapy in harmonizing both skeletal and neuromuscular parameters in growing patients.

### 3.5 Case 5—D.I., female, 6.5 years

Dentition stage: Early mixed dentition.

Diagnosis: Maxillary transverse deficiency with posterior constriction and functional Class I tendency.

Appliance: AMCOP® Integral OS; worn 1 h per day plus every night, for a total of 12 months.

#### 3.5.1 Radiographic assessment

**Figure 20:**
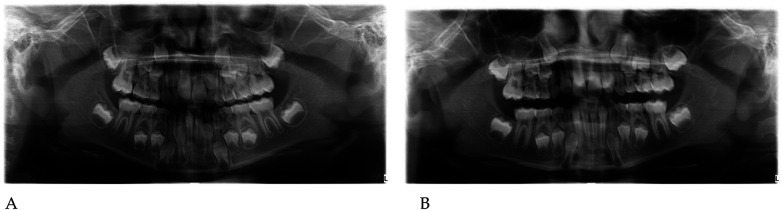
Pre- **(A)** and post-treatment **(B)** panoramic radiographs of case 5.

Panoramic radiographs (Figure 20) confirmed symmetrical dental development and absence of structural anomalies.

The eruption sequence of permanent incisors and first molars was appropriate for the patient's age, with no skeletal or dental asymmetries observed after treatment.

#### 3.5.2 Digital model analysis (palatal view)

**Figure 21:**
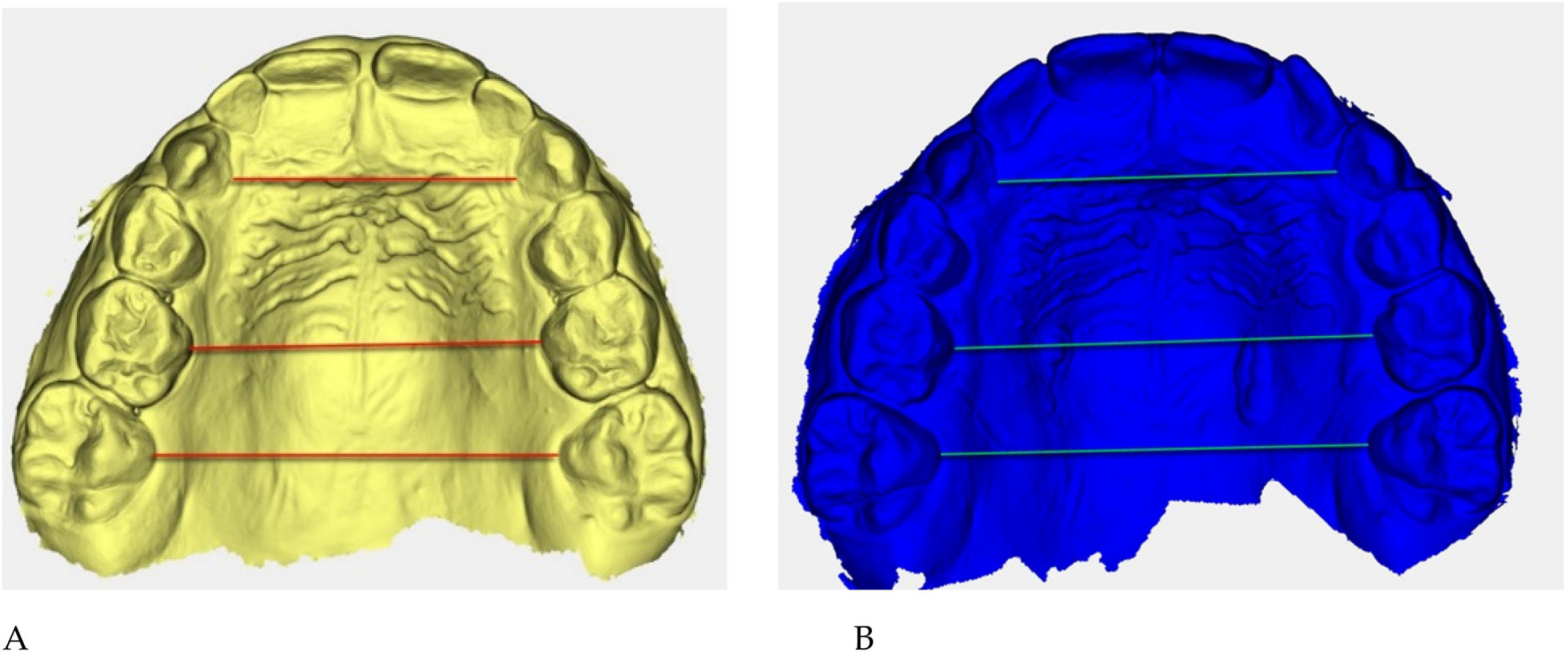
Digital 3D models of case 5 [**(A)**: pre-treatment; **(B)**: post-treatment]. Yellow = pre-treatment; blue = post-treatment. Reference lines show measured transverse distances.

Digital models acquired using *Deltadent®* software demonstrated significant transverse expansion in both the anterior and posterior regions, with a slightly higher posterior gain (Table 11, Figure 21).

**Table 11:** Transverse measurements on digital models (yellow = pre-treatment; blue = post-treatment). Δ = absolute change (post−pre); Δ% = percent change relative to pre-treatment.

Distance	Pre (mm)	Post (mm)	Δ (mm)	Δ %
Intercanine (C–C)	23.92	28.65	+4.73	+19.8%
Inter–second deciduous molars (5–5)	29.01	35.19	+6.18	+21.3%
Inter–first molars (6–6)	33.72	36.12	+2.40	+7.1%

#### 3.5.3 Clinical interpretation

After 12 months of AMCOP® Integral OS therapy, the patient achieved showed a trend toward improved symmetry across the entire maxillary arch.

The intercanine gain (+4.7 mm) and intermolar gain (+6.2 mm) indicate effective anterior and posterior remodeling, consistent with the appliance's biomechanical design.

A minor posterior predominance was noted, suggesting a balanced skeletal and functional response.

#### 3.5.4 Digital bite evaluation

**Figure 22:**
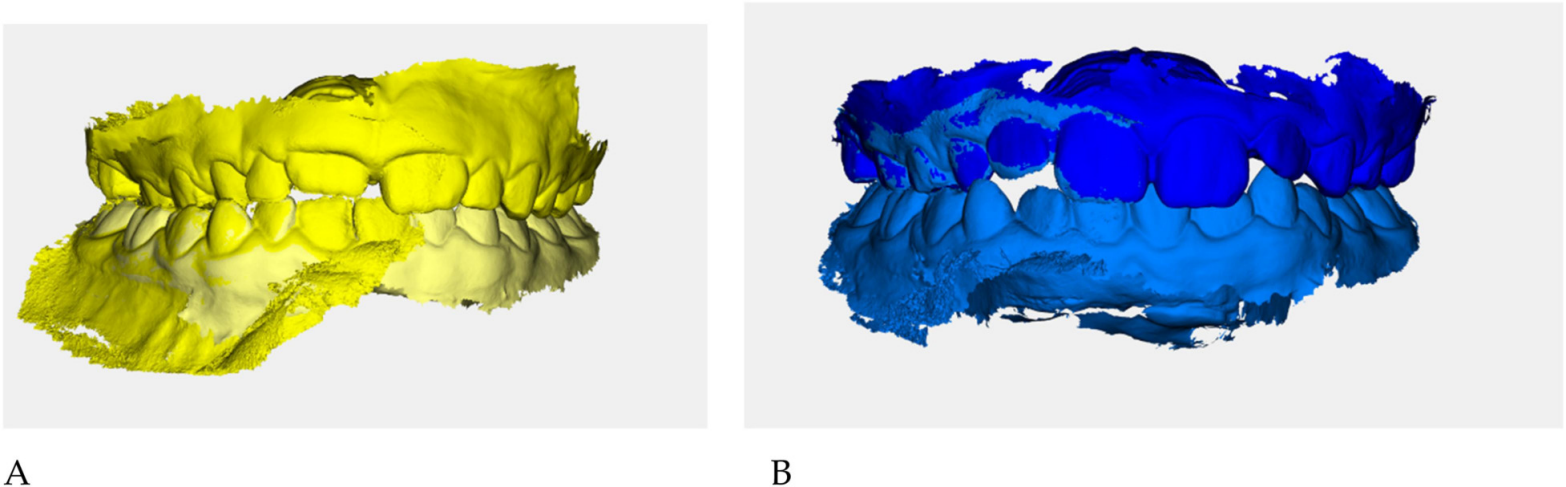
Digital occlusal models at pre- **(A)** and post-treatment **(B)**. Pre-treatment models **(A)** are color-coded in yellow (darker = maxillary, lighter = mandibular); post-treatment models **(B)** are in blue.

Color-coded occlusal models (Figures 22A,B) confirmed improved arch symmetry, enhanced transverse dimension, and better occlusal interdigitation following elastodontic therapy.

The post-treatment occlusal view demonstrated a stable arch shape and proper coordination between upper and lower arches.

#### 3.5.5 Surface electromyography (Teethan®) assessment

Neuromuscular evaluation with *Teethan® sEMG* was conducted before and after AMCOP® therapy.

**Figure 23:**
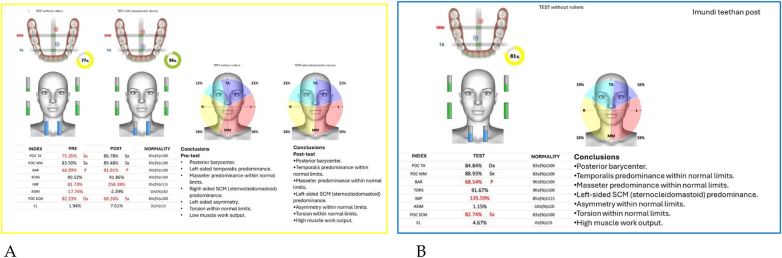
Teethan® surface electromyography. **(A)** Pre-treatment (without rollers), **(B)** post-treatment (without rollers), **(C)** post-test with elastodontic device. Post-treatment data indicate enhanced neuromuscular activation and reduced asymmetry, with improved barycenter distribution and temporalis–masseter balance.

The pre-treatment test revealed a posterior barycenter with left-side temporalis predominance and reduced global muscle efficiency. At the end of treatment, symmetry and coordination markedly improved, as shown in Table 12 and Figures 23A–C.

**Table 12:** sEMG functional indices before and after AMCOP® integral OS therapy.

Index	Pre	Post	Outcome
POC TA	75.35%	84.84%	From below → within range
POC MM	83.50%	88.93%	Improved symmetry
BAR	66.99%	68.54%	Slight posterior shift, stable
TORS	90.52%	91.67%	Within normal limits
IMP	81.73%	135.59%	Increased muscle efficiency
ASIM	−17.76%	1.15%	Restored symmetry
POC SCM	82.23%	82.74%	Stable within normal range
CL	1.94%	4.67%	Within normal range

#### 3.5.6 Interpretation

At 12 months of follow-up, the patient exhibited:

- Increases in transverse skeletal dimensions anterior +4.7 mm, posterior +6.2 mm;
- Restoration of transverse arch symmetry and proper occlusal coordination;
- Improved neuromuscular function, with normalization of POC indices and increased global muscle efficiency (IMP 135.6%);
- Stable barycenter and balanced temporalis–masseter activation.

The overall morphometric and sEMG findings were associated with favorable transverse and neuromuscular changes observed following AMCOP® elastodontic therapy in early mixed dentition.

### 3.6 Case 6—E.V., male, 7 years

Dentition stage: Early mixed dentition.

Diagnosis: Maxillary transverse deficiency with anterior constriction and functional imbalance.

Appliance: AMCOP® Integral S; worn 1 h per day plus every night, for a total of 14 months.

#### 3.6.1 Radiographic assessment

**Figure 24:**
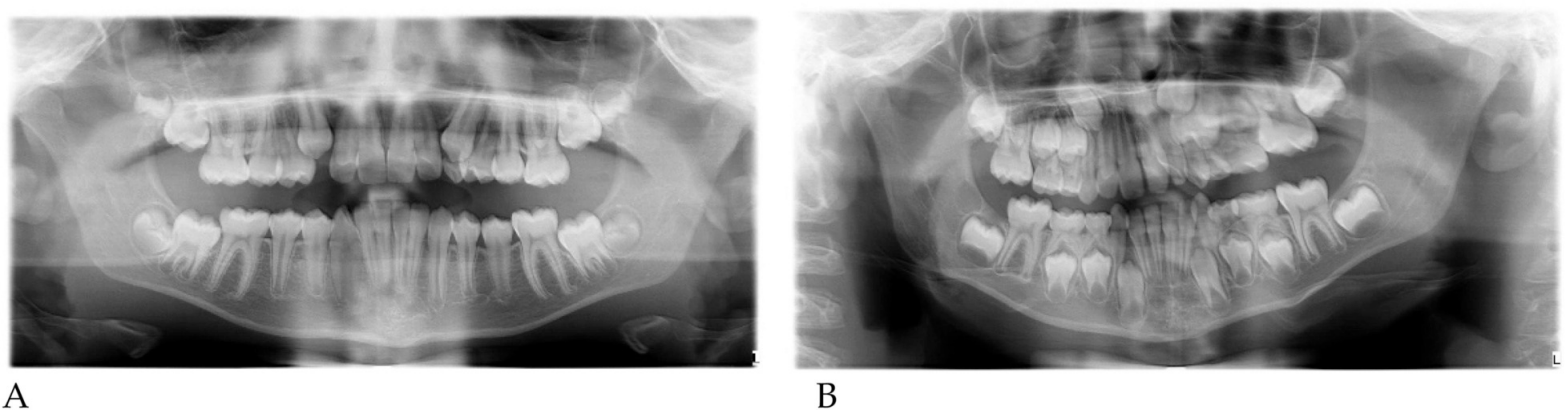
Pre- **(A)** and post-treatment **(B)** panoramic radiographs of case 6.

Panoramic radiographs (Figure 24) revealed symmetrical dental development and absence of pathologic findings.

Post-treatment imaging confirmed improved maxillary width and adequate space for the eruption of permanent teeth.

#### 3.6.2 Digital model analysis (palatal view)

Digital 3D models were analyzed using *Deltadent®* software to assess transverse dimensional changes.

**Figure 25:**
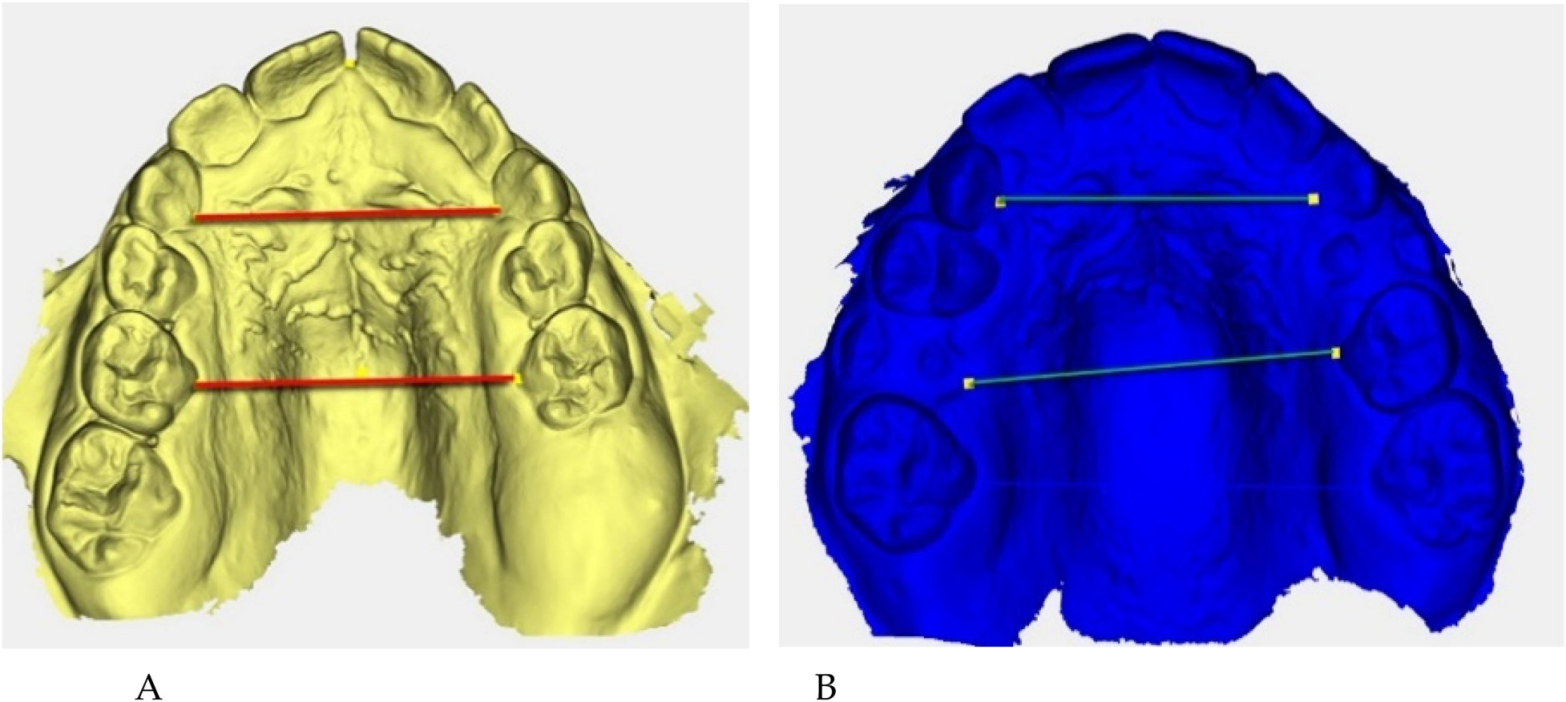
Digital 3D models of case 6 [**(A)**: pre-treatment; **(B)**: post-treatment]. Yellow = pre-treatment; blue = post-treatment. Colored reference lines indicate measured transverse distances.

The expansion pattern was more pronounced posteriorly, with significant improvement at the level of the deciduous molars (Table 13, Figure 25).

**Table 13:** Transverse measurements on digital models (yellow = pre-treatment; blue = post-treatment). Δ = absolute change (post−pre); Δ% = percent change relative to pre-treatment.

Distance	Pre (mm)	Post (mm)	Δ (mm)	Δ %
Intercanine (C–C)	23.35	24.33	+0.98	+4.2%
Inter–second deciduous molars (5–5)	25.13	28.70	+3.57	+14.2%

#### 3.6.3 Clinical interpretation

The patient showed a modest anterior expansion (+1 mm) and a greater posterior gain (+3.6 mm), indicating a physiologic, fan-shaped widening of the maxilla.

This pattern reflects the expected biomechanical response to the *Integral S* device, aimed at promoting posterior development and transverse symmetry.

#### 3.6.4 Digital bite evaluation

**Figure 26:**
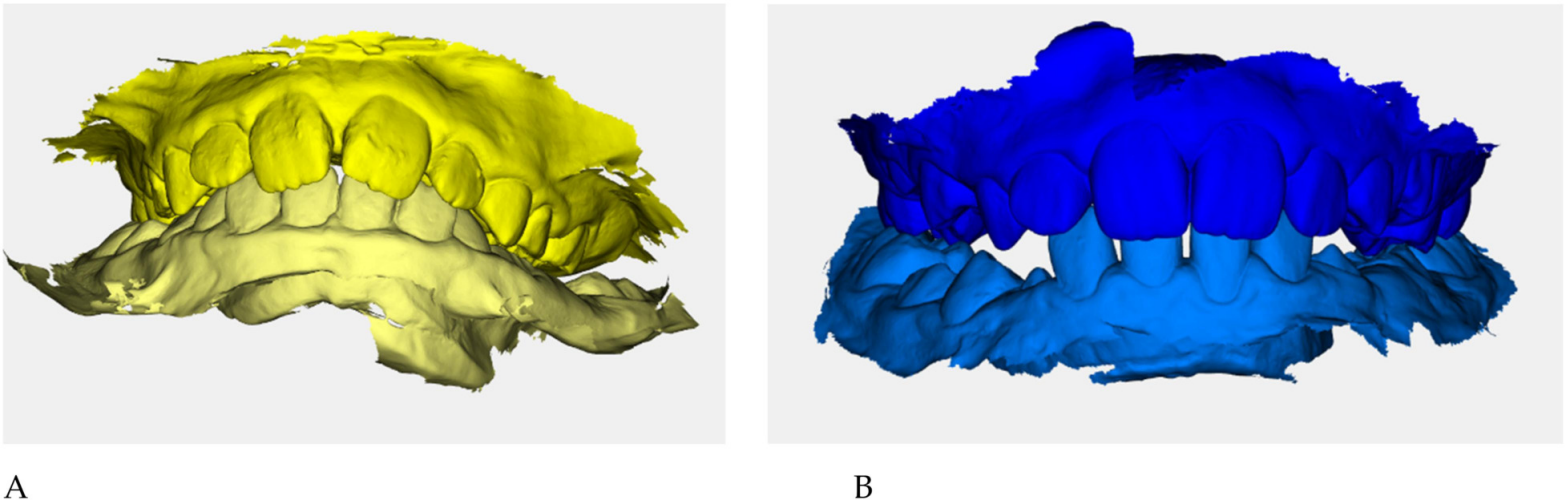
Digital occlusal models at pre- **(A)** and post-treatment **(B)**. Pre-treatment models **(A)** are color-coded in yellow (darker = maxillary, lighter = mandibular); post-treatment models **(B)** are in blue.

Color-coded occlusal digital models (Figures 26A,B) confirmed improved coordination between maxillary and mandibular arches, with increased posterior transverse dimension and more stable occlusal interdigitation.

#### 3.6.5 Surface electromyography (Teethan®) assessment

sEMG evaluation was performed before and after 14 months of elastodontic therapy.

At baseline, muscle recruitment was asymmetric, with a markedly anterior barycenter and low global muscle efficiency.

**Figure 27:**
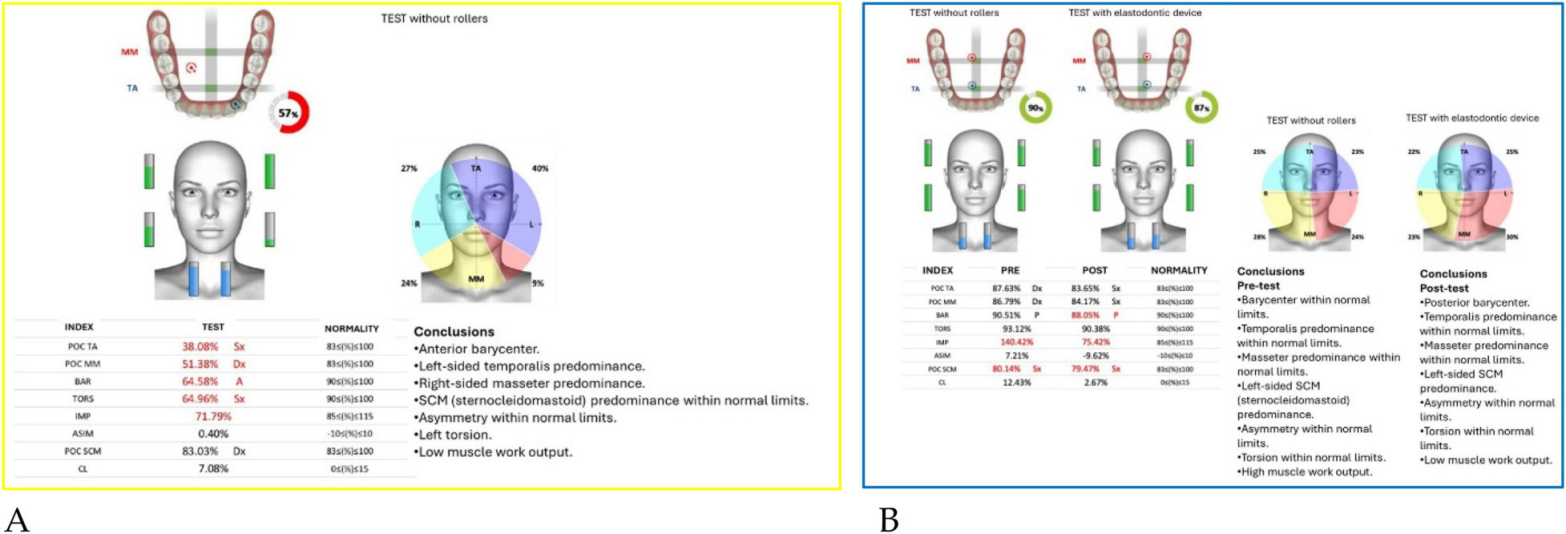
Teethan® surface electromyography. **(A)** Pre-treatment (without rollers), **(B)** post-treatment (without rollers) and with elastodontic device. After therapy, a significant increase in muscle efficiency and normalization of barycenter position were recorded.

Post-treatment data revealed normalized activation patterns, improved symmetry, and balanced temporalis–masseter coordination (Table 14, Figures 27A–C).

**Table 14:** sEMG functional indices before and after AMCOP® integral S therapy.

Index	Pre	Post	Outcome
POC TA	38.08%	87.63%	From below → normalized
POC MM	51.38%	86.79%	From below → normalized
BAR	64.58%	90.51%	Normalized (anterior → central)
TORS	64.96%	93.12%	Normalized
IMP	71.79%	140.42%	Marked improvement
ASIM	0.40%	7.21%	Within normal limits
POC SCM	83.03%	80.14%	Stable, within range
CL	7.08%	12.43%	Within normal limits

#### 3.6.6 Interpretation

After 14 months of AMCOP® Integral S therapy, the patient exhibited:

- Posteriorly predominant transverse expansion (+3.6 mm at deciduous molars);
- Improved arch coordination and occlusal stability;
- Marked neuromuscular improvement, with normalization of temporalis–masseter symmetry and restoration of balanced barycenter position;
- Increased muscle efficiency (IMP +68%), reflecting better recruitment quality.

These findings confirm that the AMCOP® Integral S appliance effectively supported both skeletal and functional balance in mixed dentition.

### 3.7 Case 7—A.D.V., male, 7 years

Dentition stage: Early mixed dentition.

Diagnosis: Maxillary transverse deficiency with mild posterior constriction.

Appliance: AMCOP® Integral S; worn 1 h per day plus every night, for a total of 12 months.

#### 3.7.1 Radiographic assessment

**Figure 28:**
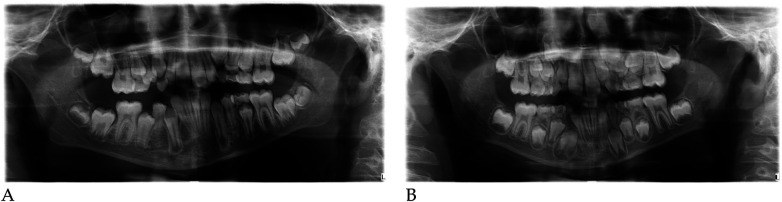
Pre- **(A)** and post-treatment **(B)** panoramic radiographs of case 7.

Panoramic radiographs (Figure 28) revealed normal dental and skeletal development for age.

No asymmetries or eruption disturbances were observed at baseline or after treatment.

Post-treatment evaluation confirmed an increased maxillary width and adequate spacing for the eruption of permanent teeth.

#### 3.7.2 Digital model analysis (palatal view)

Digital 3D models acquired with *Deltadent®* software were analyzed for transverse changes.

**Figure 29:**
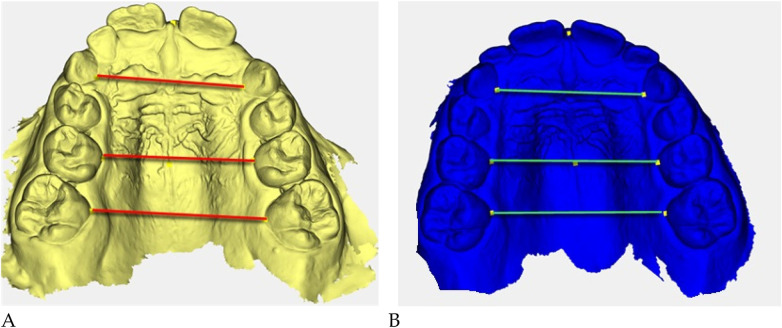
Digital 3D models of case 7 [**(A)**: pre-treatment; **(B)**: post-treatment]. Yellow = pre-treatment; blue = post-treatment. Colored reference lines correspond to inter-dental measurements used for quantitative analysis.

Quantitative data demonstrated mild but uniform expansion across the entire maxillary arch (Table 15, Figure 29).

**Table 15:** Transverse measurements on digital models (yellow = pre-treatment; blue = post-treatment). Δ = absolute change (post−pre); Δ% = percent change relative to pre-treatment.

Distance	Pre (mm)	Post (mm)	Δ (mm)	Δ %
Intercanine (C–C)	23.22	24.71	+1.49	+6.4%
Inter-premolar (5–5)	26.82	27.97	+1.15	+4.3%
Intermolar (6–6)	27.51	29.34	+1.83	+6.6%

#### 3.7.3 Clinical interpretation

After 12 months of AMCOP® Integral S therapy, the patient exhibited mild but consistent transverse gain in all regions of the arch, with the greatest widening at the molar level (+1.8 mm).

This pattern reflects a balanced expansion of the maxillary base with preservation of arch symmetry and improved coordination of occlusal planes.

#### 3.7.4 Digital bite evaluation

**Figure 30:**
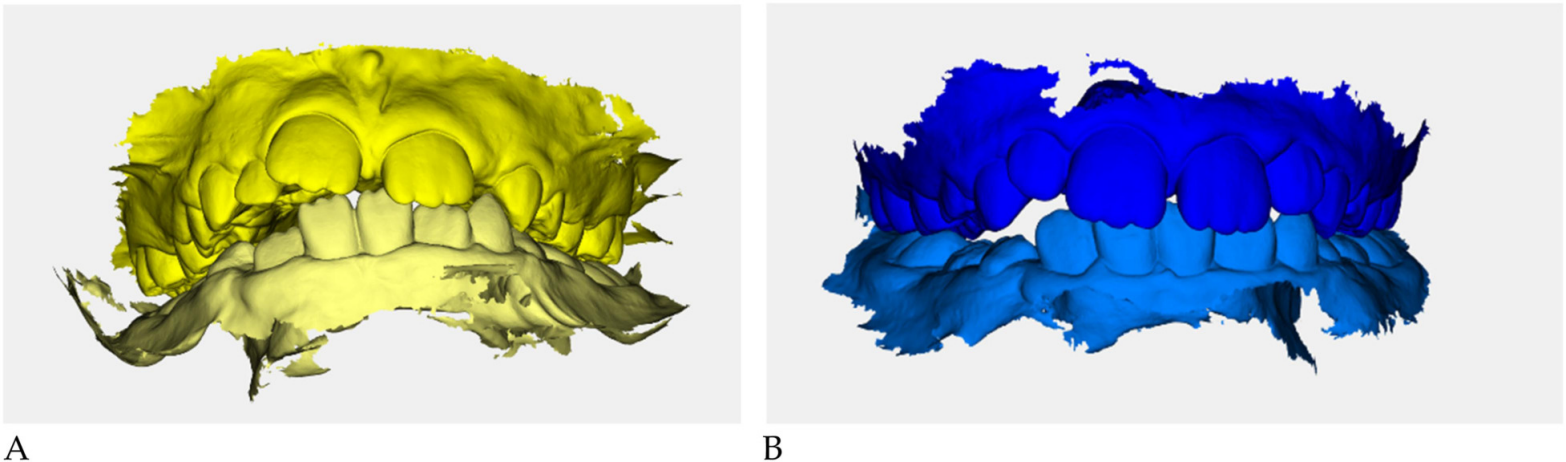
Digital occlusal models at pre- **(A)** and post-treatment **(B)**. Pre-treatment models **(A)** are color-coded in yellow (darker = maxillary, lighter = mandibular); post-treatment models **(B)** are in blue. The visualization highlights improved arch coordination and occlusal symmetry.

Color-coded occlusal digital models (Figures 30A,B) showed enhanced coordination between the upper and lower arches, with improvement in posterior intercuspation and a more stable transverse relationship following therapy.

#### 3.7.5 Surface electromyography (Teethan®) assessment

Neuromuscular analysis with *Teethan® sEMG* was performed before and after AMCOP® therapy.

The baseline test revealed anterior barycenter displacement and right-sided muscular predominance (temporalis and SCM), with low overall muscle efficiency.

**Figure 31:**
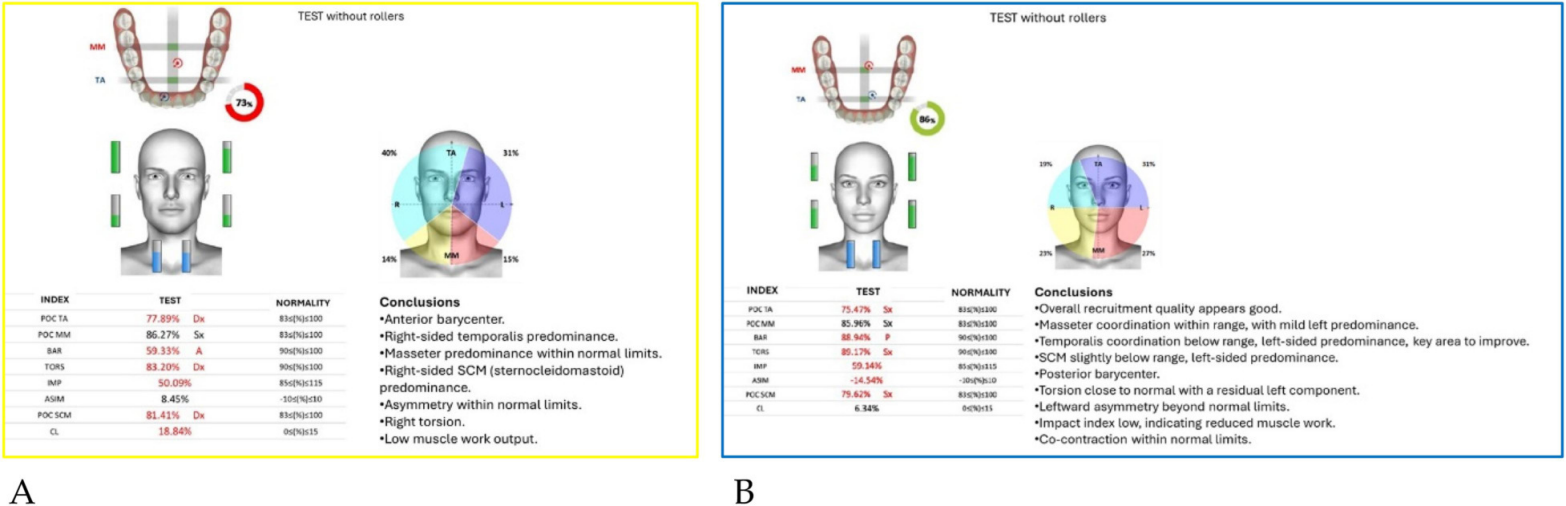
Teethan® surface electromyography. **(A)** Pre-treatment (without rollers); **(B)** post-treatment (without rollers). Post-therapy evaluation demonstrated normalization of barycenter position and improved temporalis–masseter balance, with overall enhancement in neuromuscular coordination.

After treatment, barycenter repositioning and improved bilateral coordination were observed, as detailed in Table 16 and Figures 31A,B.

**Table 16:** sEMG functional indices before and after AMCOP® integral S therapy.

Index	Pre	Post	Outcome
POC TA	77.89%	75.47%	Stable, within range
POC MM	86.27%	85.96%	Stable, within range
BAR	59.33%	88.94%	Normalized (anterior → posterior)
TORS	83.20%	89.17%	Normalized
IMP	50.09%	59.14%	Improved efficiency
ASIM	8.45%	−14.54%	Symmetry restored
POC SCM	81.41%	79.62%	Within normal limits
CL	18.84%	6.34%	Normalized

#### 3.7.6 Interpretation

After 12 months of AMCOP® Integral S therapy, the patient showed:

- Mild but consistent transverse gain across all maxillary regions (+1.1–1.8 mm);
- Improved occlusal stability and coordination between arches;
- Neuromuscular improvement, with normalization of barycenter position, reduction of asymmetry indices, and enhanced muscle efficiency (IMP +9%).

The results support the role of AMCOP® Integral S therapy in guiding physiological maxillary development and promoting functional balance in early mixed dentition.

### 3.8 Case 8—M.M., female, 5 years

Dentition stage: Early mixed dentition.

Diagnosis: Maxillary transverse deficiency with mild anterior constriction.

Appliance: AMCOP® TC; worn 1 h per day plus every night, for a total of 18 months.

#### 3.8.1 Radiographic assessment

**Figure 32:**
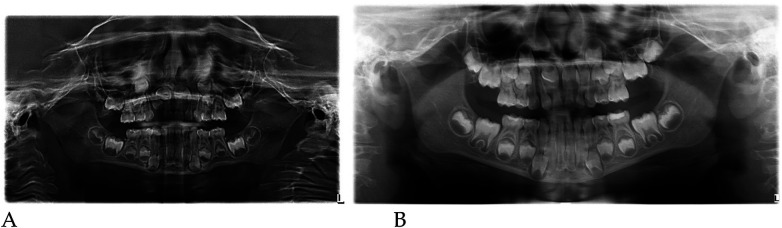
Pre- **(A)** and post-treatment **(B)** panoramic radiographs of case 8.

Panoramic radiographs (Figure 32) confirmed a regular dental development for age and symmetry of skeletal structures.

No signs of asymmetry, rotation, or delayed eruption were observed after treatment.

#### 3.8.2 Digital model analysis (palatal view)

Digital 3D models were analyzed using *Deltadent®* software to quantify the transverse expansion of the maxillary arch.

**Figure 33:**
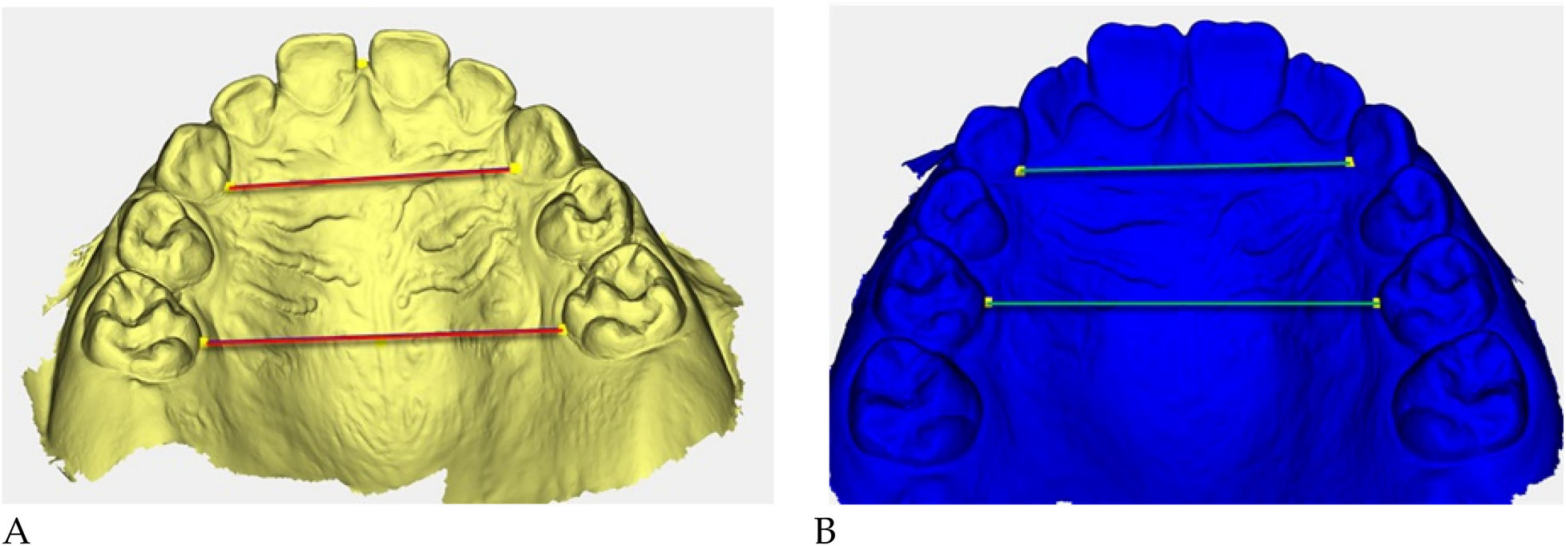
Digital 3D models of case 8 [**(A)**: pre-treatment; **(B)**: post-treatment]. Yellow = pre-treatment; blue = post-treatment. Transverse reference lines illustrate the measured intercanine and intermolar distances.

Substantial anterior and posterior gains were recorded, indicating a broad and homogeneous maxillary response to treatment (Table 17, Figure 33.

**Table 17:** Transverse measurements on digital models (yellow = pre-treatment; blue = post-treatment). Δ = absolute change (post−pre); Δ% = percent change relative to pre-treatment.

Distance	Pre (mm)	Post (mm)	Δ (mm)	Δ %
Intercanine (C–C)	21.99	26.91	+4.92	+22.4%
Inter–second deciduous molars (5–5)	27.46	31.60	+4.14	+15.1%

#### 3.8.3 Clinical interpretation

The patient exhibited significant expansion, more pronounced in the anterior region (+4.9 mm intercanine vs. + 4.1 mm posterior), resulting in an overall widening of the upper arch and a more favorable anterior corridor.

The magnitude of the increase suggests both a functional and skeletal contribution of the AMCOP® TC appliance.

#### 3.8.4 Digital bite evaluation

**Figure 34:**
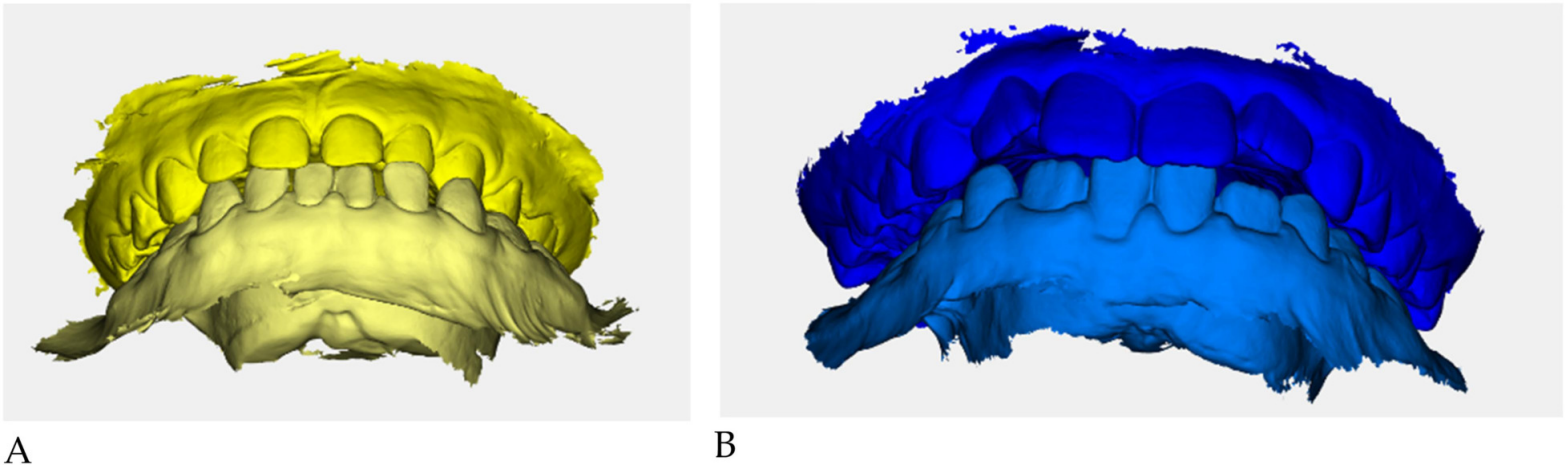
Digital occlusal models at pre- **(A)** and post-treatment **(B)**. Pre-treatment models **(A)** are color-coded in yellow (darker = maxillary, lighter = mandibular); post-treatment models **(B)** are in blue.

Color-coded digital occlusal models confirmed a marked improvement in arch symmetry and occlusal balance (Figure 34).

Post-treatment visualization revealed smoother arch curvature and greater intercuspation between maxillary and mandibular teeth.

#### 3.8.5 Surface electromyography (Teethan®) assessment

sEMG analysis was performed using *Teethan®* before and after the AMCOP® TC treatment.

At baseline, the patient presented anterior barycenter displacement, low muscle efficiency, and left-sided predominance in temporalis and masseter activation.

**Figure 35:**
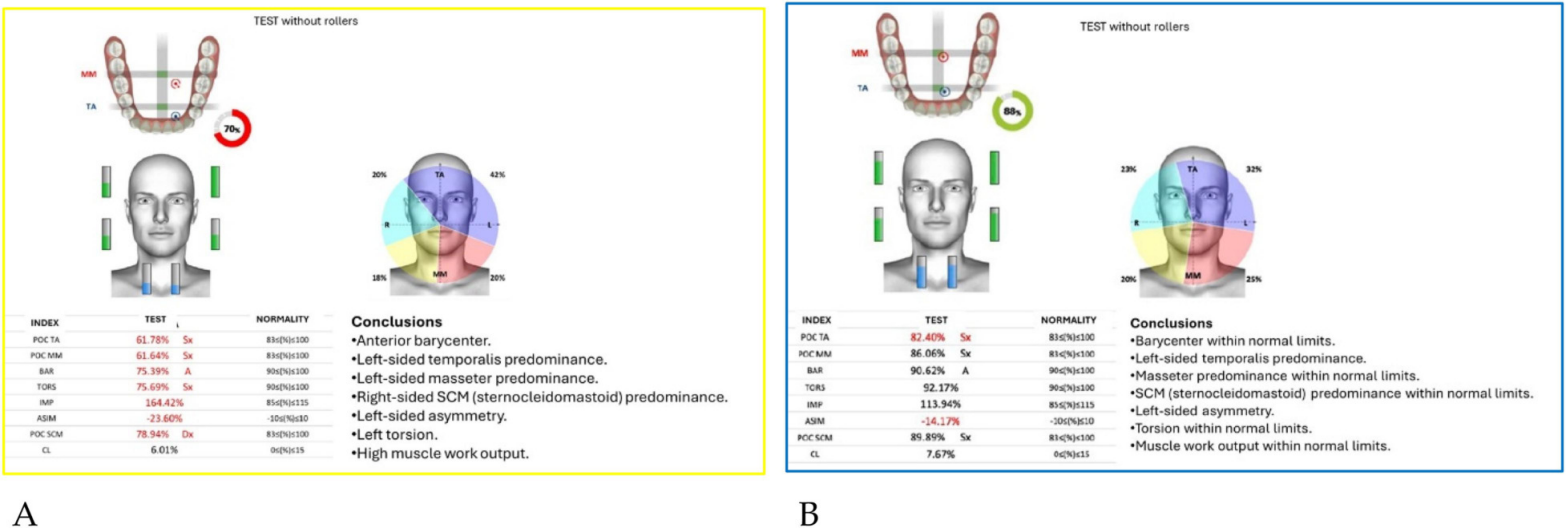
Teethan® surface electromyography. **(A)** Pre-treatment (without rollers); **(B)** post-treatment (without rollers). The post-therapy pattern shows normalization of barycenter position, balanced temporalis–masseter activity and improved overall neuromuscular coordination.

After 18 months, muscle symmetry and coordination improved substantially, as summarized in Table 18 and Figures 35A,B.

**Table 18:** sEMG functional indices before and after AMCOP® TC therapy.

Index	Pre	Post	Outcome
POC TA	61.78%	82.40%	From below → within normal limits
POC MM	61.64%	86.06%	From below → normalized
BAR	75.39%	90.62%	Normalized (anterior → central)
TORS	75.69%	92.17%	Normalized
IMP	164.42%	113.94%	From hyperactivation → normal
ASIM	−23.60%	−14.17%	Improved symmetry
POC SCM	78.94%	89.89%	Normalized
CL	6.01%	7.67%	Stable within normal range

#### 3.8.6 Interpretation

After 18 months of AMCOP® TC therapy, the patient demonstrated:

- Marked transverse expansion, particularly anteriorly (+4.9 mm), with posterior improvement (+4.1 mm);
- Restoration of arch symmetry and improved interarch coordination;
- Normalization of sEMG indices, with balanced temporalis and masseter activity;
- Improved barycenter position and reduced asymmetry;
- Overall neuromuscular optimization, consistent with efficient functional adaptation.

The combined morphometric and electromyographic outcomes confirm the efficacy of AMCOP® TC in promoting balanced skeletal and muscular development during early mixed dentition.

### 3.9 Case 9—L.L., female, 5 years

Dentition stage: Early mixed dentition.

Diagnosis: Maxillary transverse deficiency with anterior constriction and functional imbalance.

Appliance: AMCOP® TC; worn 1 h per day plus every night, for a total of 14 months.

#### 3.9.1 Radiographic assessment

**Figure 36:**
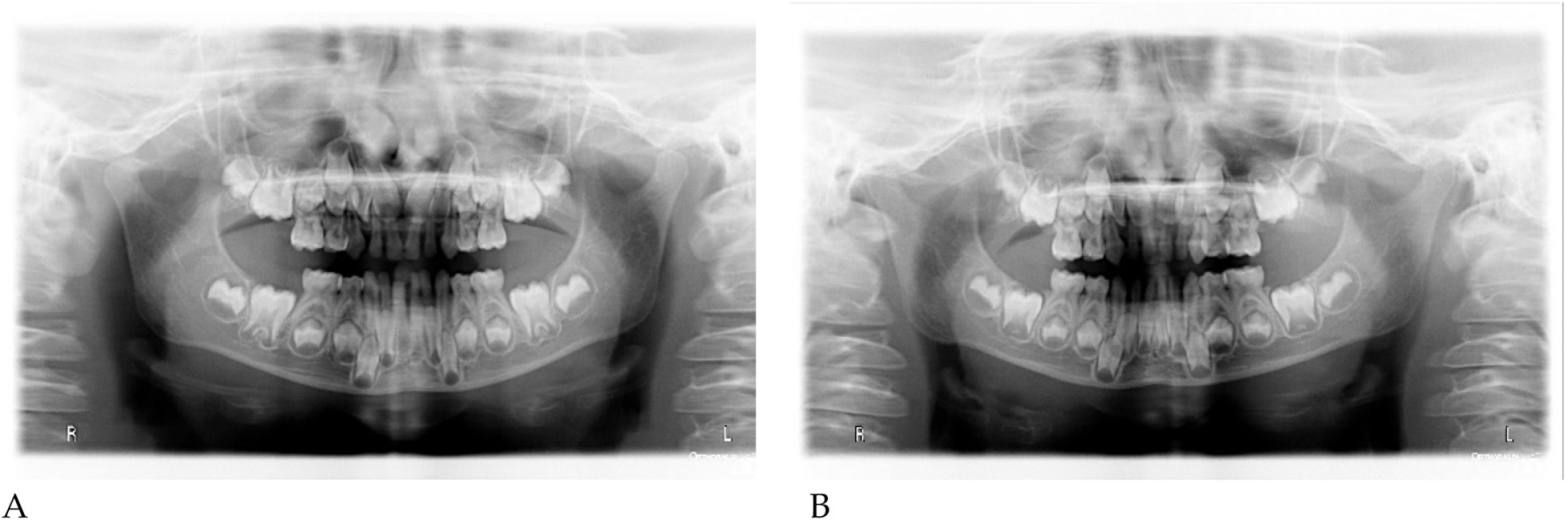
Pre- **(A)** and post-treatment **(B)** panoramic radiographs of case 9.

Panoramic radiographs (Figure 36) revealed normal root morphology and symmetric development of both dental arches.

No asymmetries or delayed eruptions were noted before or after therapy.

#### 3.9.2 Digital model analysis (palatal view)

Three-dimensional digital models were obtained and analyzed with *Deltadent®* software.

**Figure 37:**
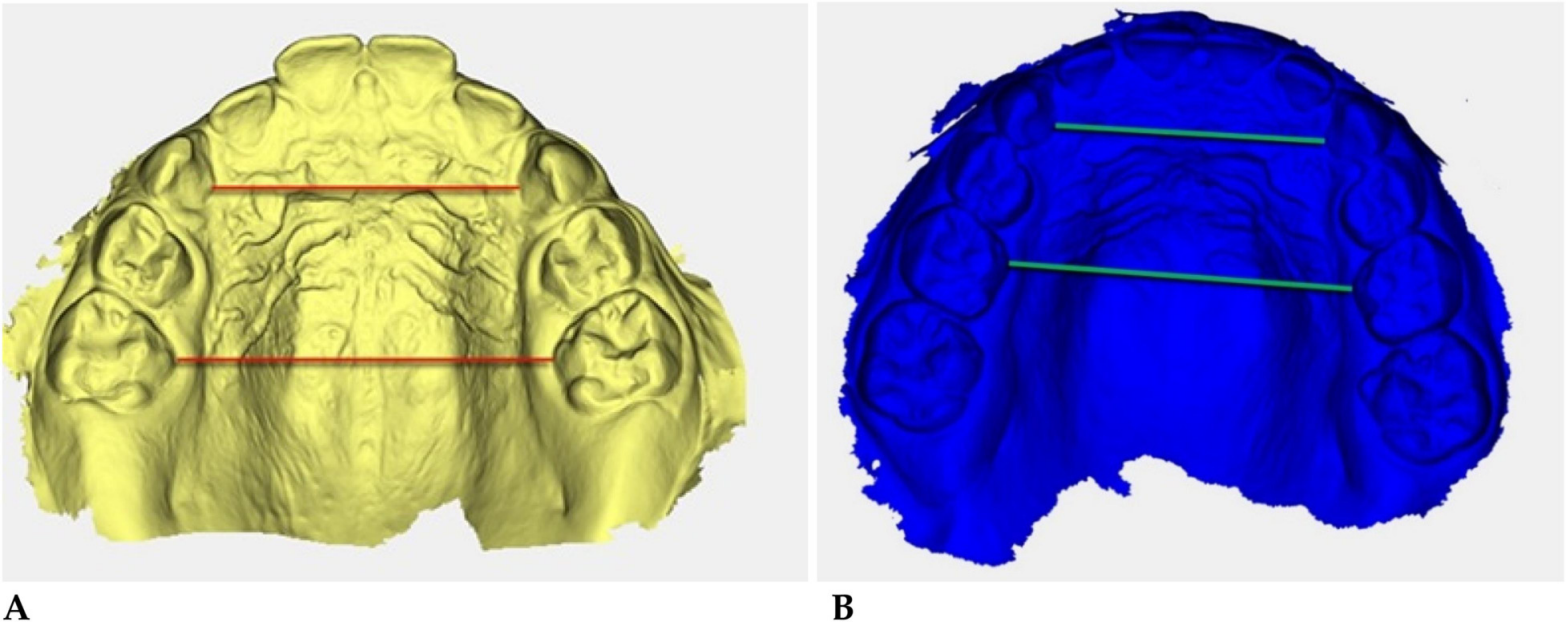
Digital 3D models of case 9 [**(A)**: pre-treatment; **(B)**: post-treatment]. Yellow = pre-treatment; blue = post-treatment. Reference lines indicate transverse measurement levels.

A mild yet consistent transverse gain was observed in both anterior and posterior regions of the maxilla (Table 19, Figure 37).

**Table 19:** Transverse measurements on digital models (yellow = pre-treatment; blue = post-treatment). Δ = absolute change (post−pre); Δ% = percent change relative to pre-treatment.

Distance	Pre (mm)	Post (mm)	Δ (mm)	Δ %
Intercanine (C–C)	22.74	23.24	+0.50	+2.2%
Inter–second deciduous molars (5–5)	27.35	29.51	+2.16	+7.9%

#### 3.9.3 Clinical interpretation

The arch displayed a predominantly posterior widening pattern, with a larger increase at the molar level (+2.2 mm) compared to the anterior region (+0.5 mm).

The result reflects a controlled expansion of the maxillary base consistent with the biomechanical action of the AMCOP® TC appliance.

#### 3.9.4 Digital bite evaluation

**Figure 38:**
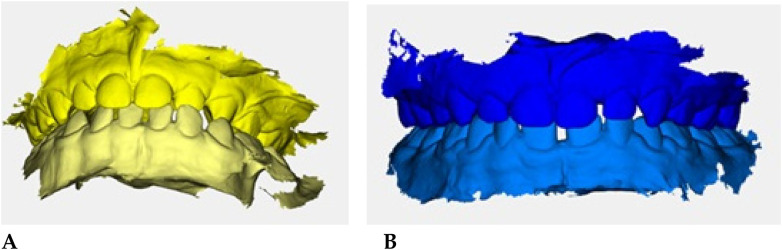
Digital occlusal models at pre- **(A)** and post-treatment **(B)** Pre-treatment models **(A)** are color-coded in yellow (darker = maxillary, lighter = mandibular); post-treatment models **(B)** are in blue.

Occlusal color-coded digital models confirmed enhanced posterior coordination and improved intercuspation, particularly in the right molar region, with balanced development of the transverse dimension (Figure 38).

#### 3.9.5 Surface electromyography (Teethan®) assessment

Surface EMG (*Teethan®*) analysis was performed before and after 14 months of AMCOP® TC therapy.

At baseline, the patient exhibited an anterior barycenter, right-sided temporalis predominance, and low overall muscle efficiency.

**Figure 39:**
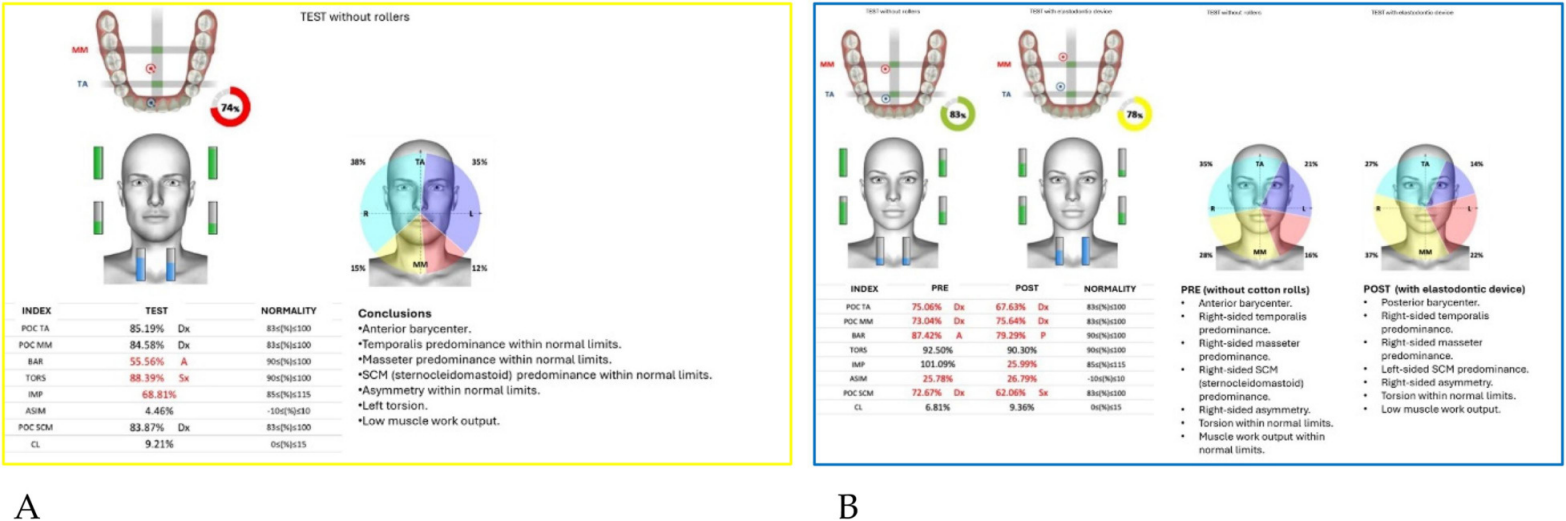
Teethan® surface electromyography. **(A)** Pre-treatment (without rollers); **(B)** post-treatment (without rollers) and with elastodontic device. The post-treatment trace showed normalization of barycenter position and improved activation symmetry, consistent with enhanced neuromuscular control.

After treatment, barycenter position improved, and muscular balance between sides was restored (Table 20, Figures 39A,B).

**Table 20:** sEMG functional indices before and after AMCOP® TC therapy.

Index	Pre	Post	Outcome
POC TA	85.19%	75.06%	Within normal limits
POC MM	84.58%	73.04%	Slight decrease, within range
BAR	55.56%	87.42%	Normalized (anterior → posterior)
TORS	88.39%	92.50%	Normalized
IMP	68.81%	101.09%	Improved muscle efficiency
ASIM	4.46%	25.78%	Minor residual asymmetry
POC SCM	83.87%	72.67%	Within acceptable range
CL	9.21%	6.81%	Normal range

#### 3.9.6 Interpretation

After 14 months of AMCOP® TC therapy, the patient demonstrated:

- Posteriorly predominant expansion, especially at the deciduous molar level (+2.16 mm);
- Improved occlusal coordination and arch symmetry;
- Neuromuscular rebalancing, with normalization of barycenter position and increased efficiency (IMP +32%);
- Functional stabilization, indicating restored temporalis–masseter coordination and improved postural muscle control.

The combined morphometric and electromyographic results confirm that AMCOP® TC therapy effectively harmonized skeletal and functional parameters, supporting stable maxillary development in early mixed dentition.

### 3.10 Case 10—A.G.V., female, 4 years

Dentition stage: Early mixed dentition (emerging first permanent molars).

Diagnosis: Maxillary transverse deficiency with reduced anterior arch width.

Appliance: AMCOP® Integral S1; worn 1 h per day plus every night, for a total of 12 months.

#### 3.10.1 Radiographic assessment

**Figure 40:**
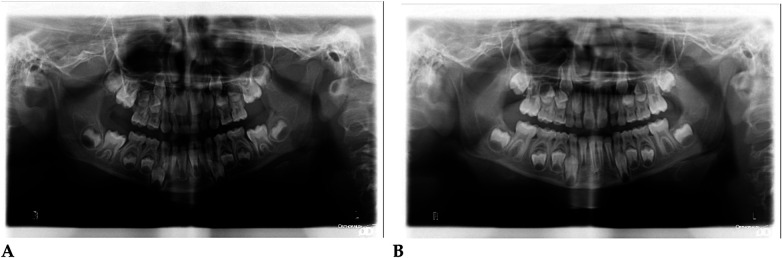
Pre- **(A)** and post-treatment **(B)** panoramic radiographs of case 10.

Panoramic radiographs (Figure 40) revealed normal eruption patterns and symmetric skeletal growth.

No pathological findings or asymmetries were detected pre- or post-treatment.

#### 3.10.2 Digital model analysis (palatal view)

Digital 3D models obtained through *Deltadent®* software were used to evaluate transverse changes.

**Figure 41:**
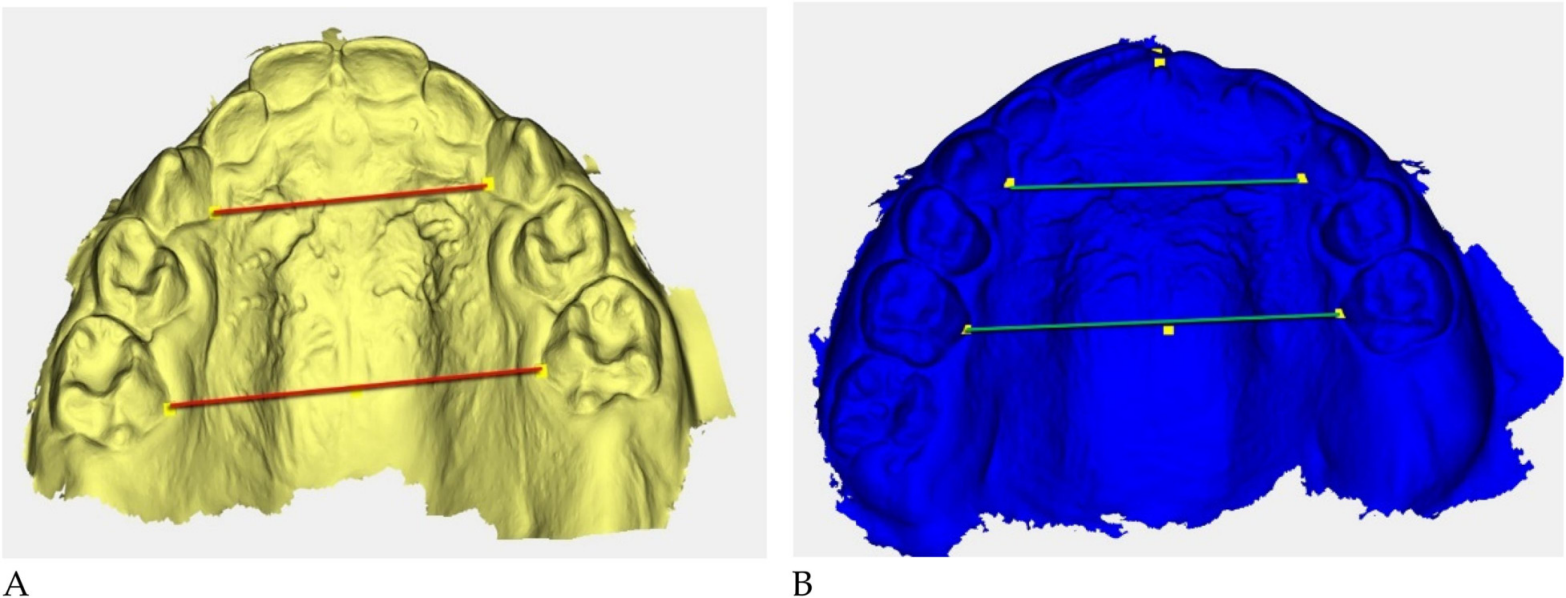
Digital 3D models of case 10 [**(A)**: pre-treatment; **(B)**: post-treatment]. Yellow = pre-treatment; blue = post-treatment. Colored guidelines represent the transverse reference points for measurement.

A marked anterior and posterior expansion was observed after therapy (Table 21, Figure 41).

**Table 21:** Transverse measurements on digital models (yellow = pre-treatment; blue = post-treatment). Δ = absolute change (post−pre); Δ% = percent change relative to pre-treatment.

Distance	Pre (mm)	Post (mm)	Δ (mm)	Δ %
Intercanine (C–C)	19.17	22.88	+3.71	+19.4%
Inter–second deciduous molars (5–5)	25.79	29.15	+3.36	+13.0%

#### 3.10.3 Clinical interpretation

The patient exhibited a harmonic anterior and posterior expansion, with slightly greater widening in the intercanine region (+3.7 mm) than posteriorly (+3.4 mm).

This pattern is consistent with the expected action of the AMCOP® S1 Integral device, which promotes anterior development and corrects transverse deficiency while maintaining arch symmetry.

#### 3.10.4 Digital bite evaluation

**Figure 42:**
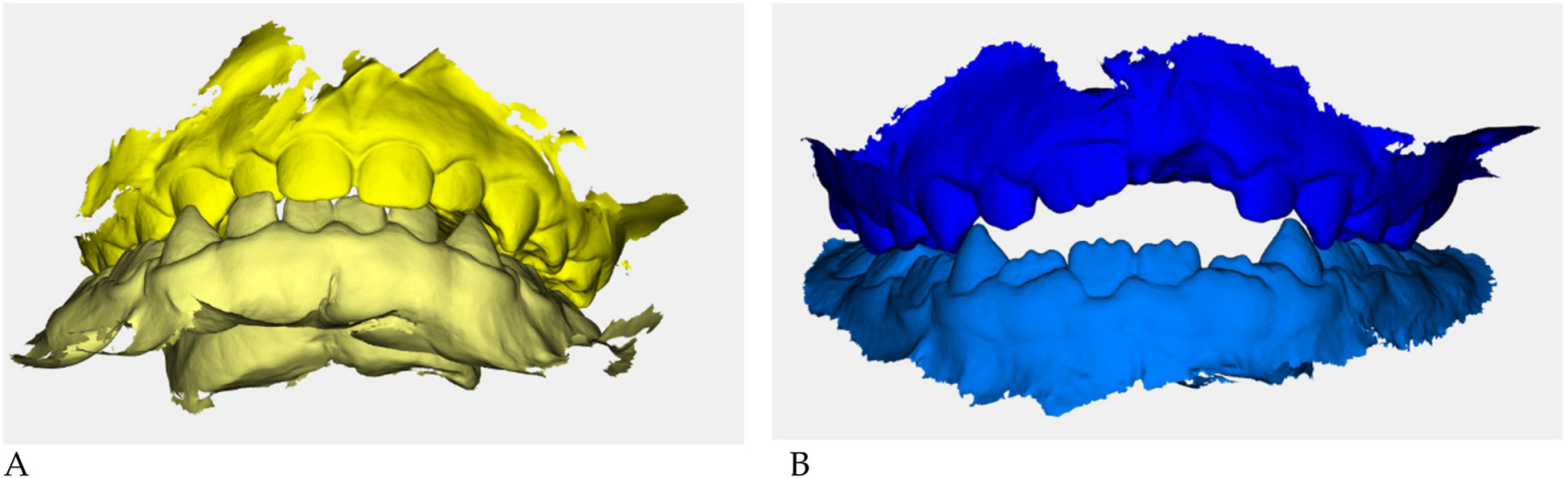
Digital occlusal models at pre- **(A)** and post-treatment **(B)**. Pre-treatment models **(A)** are color-coded in yellow (darker for the maxillary arch, lighter for mandibular); post-treatment models **(B)** are in blue. Visualization confirms improved symmetry and transverse coordination following elastodontic therapy.

Qualitative analysis of color-coded occlusal models revealed improved arch coordination, increased transverse width, and enhanced intercuspation between maxillary and mandibular arches (Figure 42).

Posterior occlusal balance improved markedly after therapy.

#### 3.10.5 Surface electromyography (Teethan®) assessment

Surface electromyographic recordings (*Teethan®*) were performed before and after AMCOP® therapy.

At baseline, the patient exhibited markedly anterior barycenter, low muscle efficiency, and asymmetric temporalis–masseter activation.

**Figure 43:**
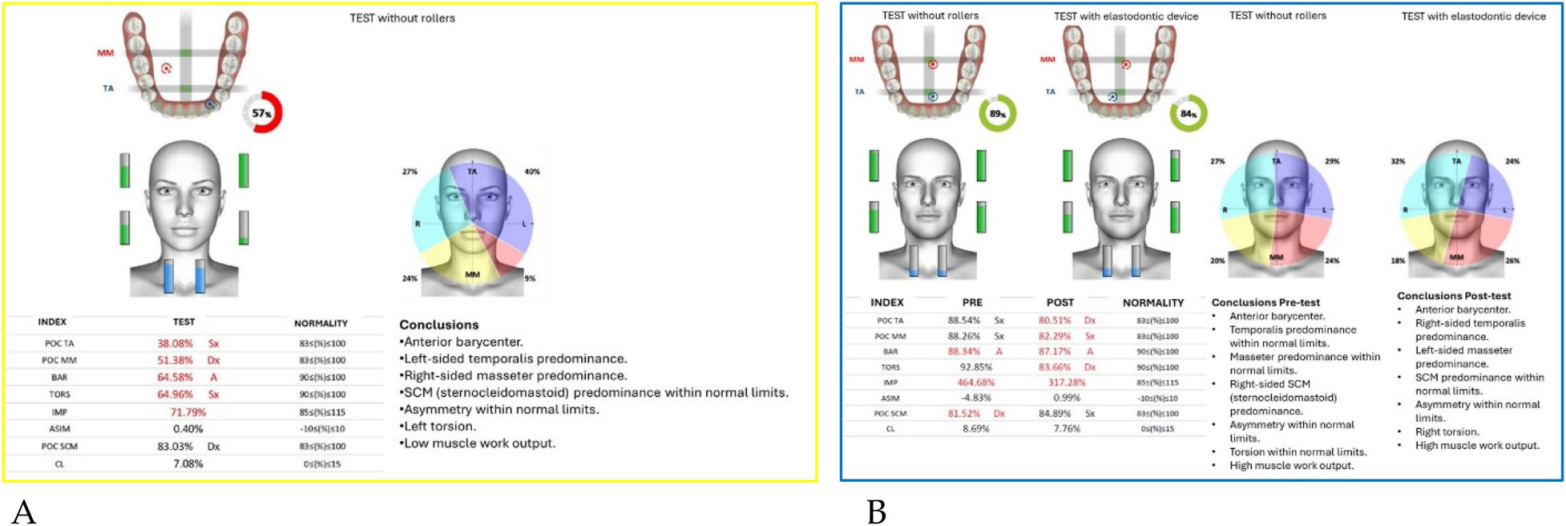
Teethan® surface electromyography. **(A)** Pre-treatment (without rollers); **(B)** post-treatment (with elastodontic device). Post-therapy traces demonstrate restored muscle balance, normalization of barycenter position, and a shift toward efficient neuromuscular recruitment.

After 12 months, a significant improvement was observed in muscle recruitment balance, despite a persistent mild right predominance (Table 22, Figures 43A,B).

**Table 22:** sEMG functional indices before and after AMCOP® S1 therapy.

Index	Pre	Post	Outcome
POC TA	38.08%	80.51%	From below → normalized
POC MM	51.38%	82.29%	From below → normalized
BAR	64.58%	87.17%	Normalized (anterior → central)
TORS	64.96%	83.66%	Improved, within normal limits
IMP	71.79%	317.28%	From low → high efficiency
ASIM	0.40%	0.99%	Stable symmetry
POC SCM	83.03%	84.89%	Within normal limits
CL	7.08%	7.76%	Normalized

#### 3.10.6 Interpretation

After 12 months of AMCOP® Integral S1 therapy, the patient showed:

- Significant transverse gain, both anteriorly (+3.7 mm) and posteriorly (+3.4 mm);
- Improved occlusal coordination and arch symmetry;
- Marked neuromuscular rebalancing, with normalization of POC indices and barycenter alignment;
- Enhanced muscle efficiency, consistent with optimal adaptation to the elastodontic correction.

Overall, the treatment led to a functional and morphological normalization of the maxillary arch and masticatory system in early mixed dentition.

### 3.11 Case 11—L.P., male, 7 years

Dentition stage: Early mixed dentition.

Diagnosis: Skeletal Class III with maxillary transverse deficiency.

Appliance: AMCOP® Class III device; worn 1 h per day plus every night, for a total of 12 months.

#### 3.11.1 Radiographic assessment

**Figure 44:**
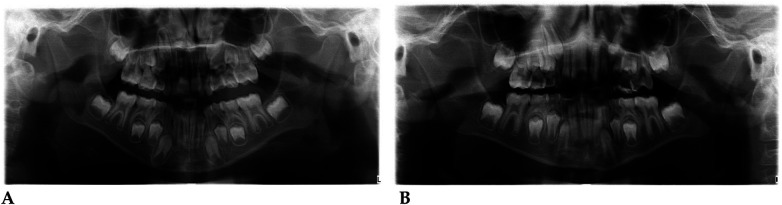
Pre- **(A)** and post-treatment **(B)** panoramic radiographs of case 11.

Panoramic evaluation (Figure 44) confirmed proper eruption timing, normal root morphology, and symmetrical skeletal development.

No anomalies or dental asymmetries were observed after treatment.

#### 3.11.2 Digital model analysis (palatal view)

Transverse maxillary dimensions were analyzed using *Deltadent®* software.

**Figure 45:**
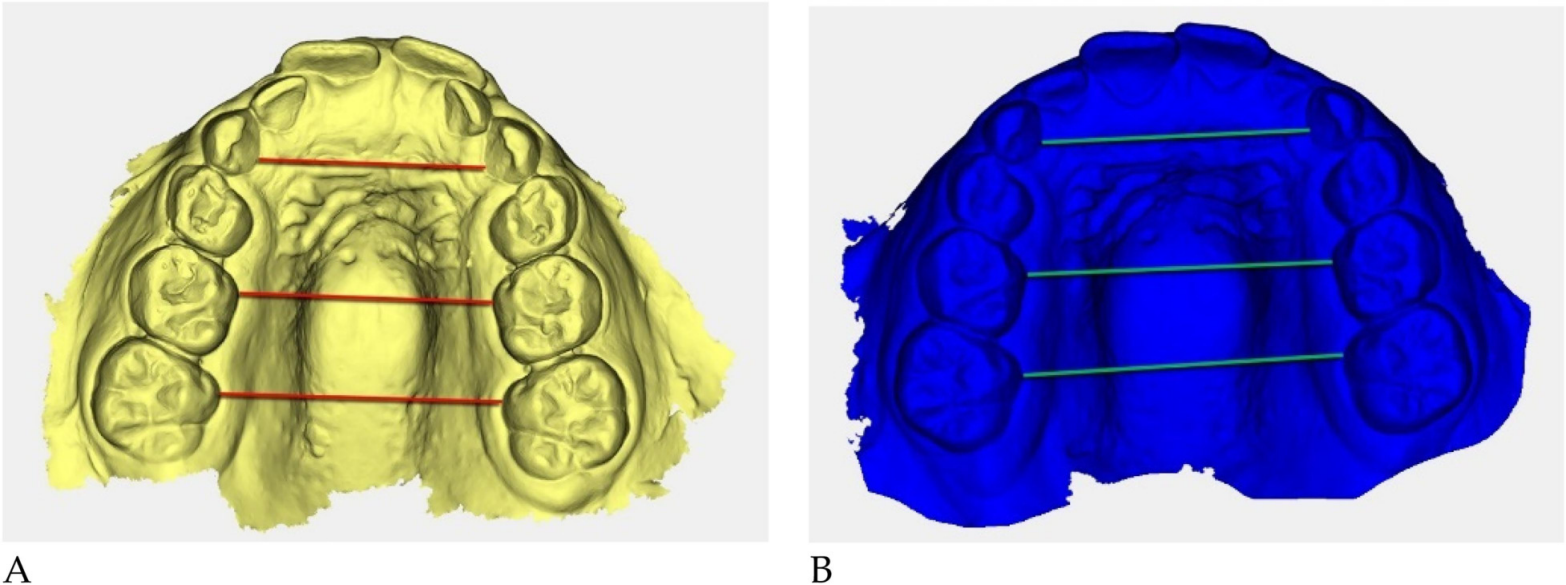
Digital 3D models of case 11 [**(A)**: pre-treatment; **(B)**: post-treatment]. Yellow = pre-treatment; blue = post-treatment. Colored reference lines highlight transverse width measurements.

A uniform anterior–posterior expansion was recorded, indicating effective correction of transverse deficiency (Table 23, Figure 45).

**Table 23:** Transverse measurements on digital models (yellow = pre-treatment; blue = post-treatment). Δ = absolute change (post−pre); Δ% = percent change relative to pre-treatment.

Distance	Pre (mm)	Post (mm)	Δ (mm)	Δ %
Intercanine (C–C)	20.50	24.72	+4.22	+20.6%
Inter–second deciduous molars (5–5)	22.52	27.95	+5.43	+24.1%
Inter–first permanent molars (6–6)	25.26	29.20	+3.94	+15.6%

#### 3.11.3 Clinical interpretation

The patient achieved a broad and harmonic maxillary expansion, greater in the premolar region (+5.4 mm), suggesting optimal stimulation of the midpalatal suture and dentoalveolar remodeling.

The intercanine gain (+4.2 mm) reflects balanced anterior development, suitable for early Class III correction.

#### 3.11.4 Digital bite evaluation

Qualitative 3D analysis demonstrated correction of anterior constriction and posterior crossbite tendency.

**Figure 46:**
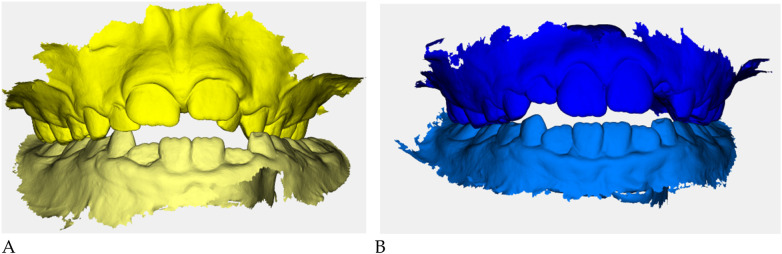
Digital occlusal models at pre- **(A)** and post-treatment **(B)**. Pre-treatment model **(A)** in yellow; post-treatment model **(B)** in blue, showing enhanced arch symmetry and occlusal integration.

The post-treatment model revealed improved transverse coordination and symmetrical intercuspation (Figure 46).

#### 3.11.5 Surface electromyography (Teethan®) assessment

Surface electromyography (*Teethan®*) was used to evaluate neuromuscular balance before and after AMCOP® therapy.

At baseline, muscle activity was within normal limits but characterized by slightly anterior barycenter and moderate asymmetry.

**Figure 47:**
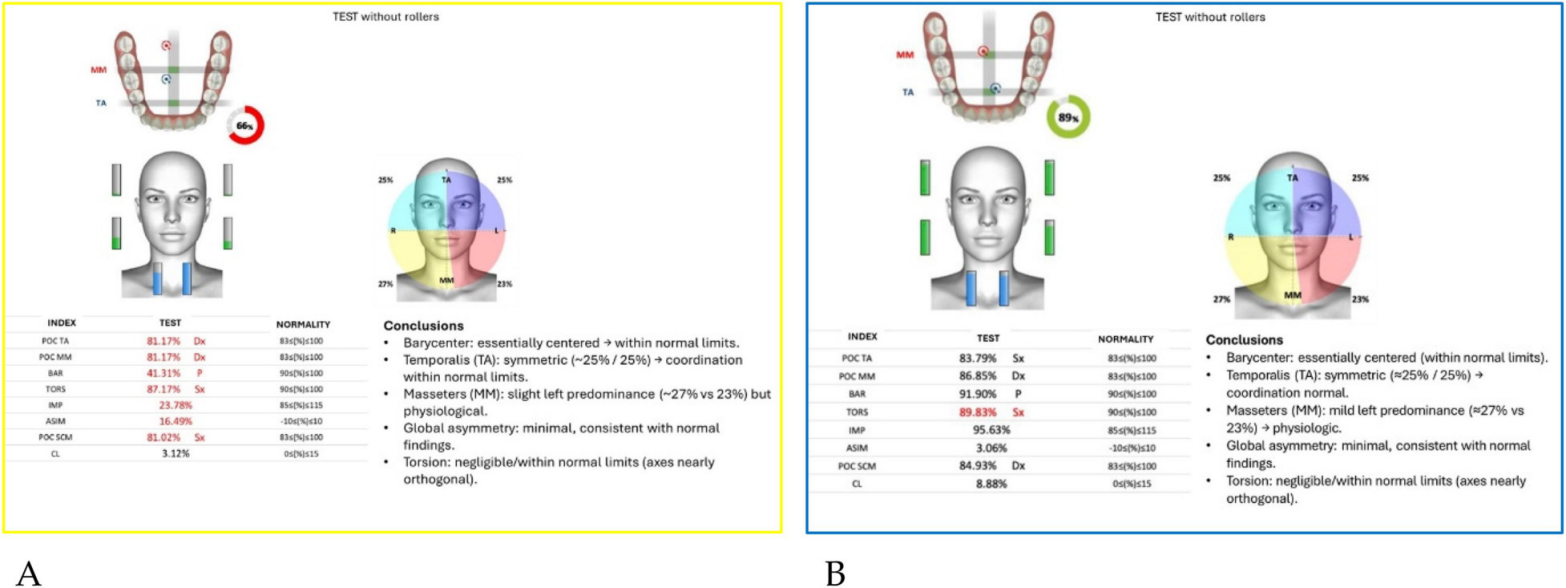
Teethan® surface electromyography. **(A)** Pre-treatment (without rollers); **(B)** post-treatment (without rollers). The post-therapy trace demonstrates restored barycenter position, enhanced temporalis and masseter coordination, and normalization of muscle efficiency.

After 12 months, a significant improvement in barycenter position and muscle coordination was detected (Table 24, Figures 47A,B).

**Table 24:** sEMG functional indices before and after AMCOP® class III therapy.

Index	Pre	Post	Outcome
POC TA	81.17%	83.79%	Normal range, mild improvement
POC MM	81.17%	86.85%	Normalized
BAR	41.31%	91.90%	Normalized (anterior → central)
TORS	87.17%	89.83%	Stable within range
IMP	23.78%	95.63%	Marked improvement
ASIM	16.49%	3.06%	Restored symmetry
POC SCM	81.02%	84.93%	Normalized
CL	3.12%	8.88%	Within range

#### 3.11.6 Interpretation

After 12 months of AMCOP® Class III therapy, the patient demonstrated:

- Marked transverse expansion, especially at the premolar level (+5.4 mm);
- Functional correction of anterior maxillary constriction;
- Normalization of barycenter position and muscle symmetry;
- Improved neuromuscular efficiency (IMP ↑ from 23.8% → 95.6%);
- Stable occlusal and muscular balance, consistent with functional adaptation.

These findings describe transverse dimensional changes and neuromuscular pattern variations observed in association with early AMCOP® Class III therapy in growing patients.

### 3.12 Case 12—G.D.D., female, 7 years

Dentition stage: Early mixed dentition.

Diagnosis: Maxillary transverse deficiency with mild anterior constriction and neuromuscular imbalance.

Appliance: AMCOP® OS Integral; worn 1 h per day plus every night, for a total of 14 months.

#### 3.12.1 Radiographic assessment

**Figure 48:**
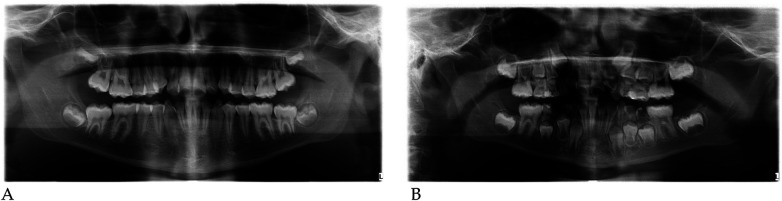
Pre- **(A)** and post-treatment **(B)** panoramic radiographs of case 12.

Panoramic radiographs (Figure 48) revealed a regular eruption sequence, normal root morphology, and symmetric skeletal growth.

No dental anomalies were detected before or after treatment.

#### 3.12.2 Digital model analysis (palatal view)

**Figure 49:**
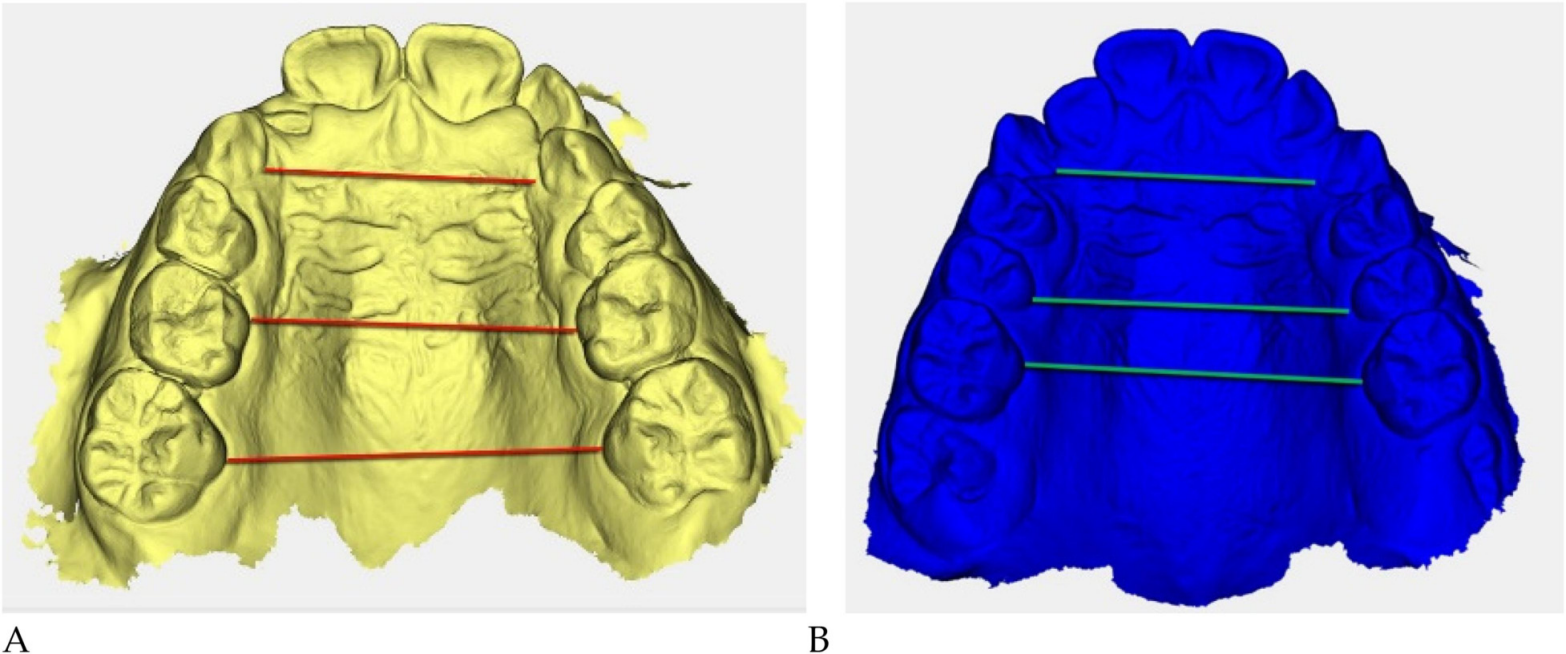
Digital 3D models of case 12 [**(A)**: pre-treatment; **(B)**: post-treatment]. Yellow = pre-treatment; blue = post-treatment. Reference lines indicate the measurement planes for transverse analysis.

Three-dimensional *Deltadent®* analysis showed a marked improvement in transverse maxillary dimensions following AMCOP® OS Integral therapy (Table 25, Figure 49).

**Table 25:** Transverse measurements on digital models (yellow = pre-treatment; blue = post-treatment). Δ = absolute change (post−pre); Δ% = percent change relative to pre-treatment.

Distance	Pre (mm)	Post (mm)	Δ (mm)	Δ %
Intercanine (C–C)	21.97	25.86	+3.89	+17.7%
Inter–second deciduous molars (5–5)	26.30	31.21	+4.91	+18.7%
Inter–first permanent molars (6–6)	29.59	33.49	+3.90	+13.2%

#### 3.12.3 Clinical interpretation

The digital analysis demonstrates uniform maxillary widening, with the greatest transverse gain observed in the premolar region (+4.9 mm).

This reflects a harmonic skeletal and dentoalveolar response, typical of AMCOP® OS Integral therapy, which effectively corrects posterior constriction while preserving anterior arch form.

#### 3.12.4 Digital bite evaluation

Color-coded occlusal models confirmed improved transverse coordination and symmetry.

**Figure 50:**
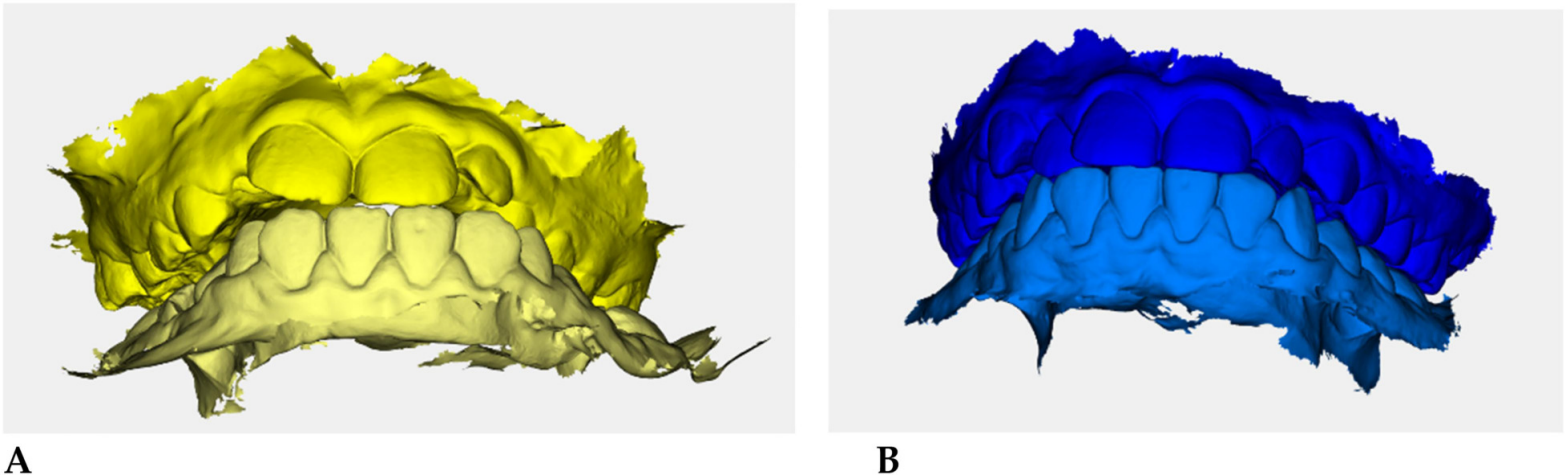
Digital occlusal models at pre- **(A)** and post-treatment **(B)**. Pre-treatment models **(A)** are shown in yellow, post-treatment models **(B)** in blue. Post-treatment changes include increased posterior width and improved occlusal interdigitation.

Post-treatment evaluation revealed normalized posterior occlusal contacts and functional alignment of the maxillary arch (Figure 50).

#### 3.12.5 Surface electromyography (Teethan®) assessment

Surface electromyography (*Teethan®*) was performed before and after AMCOP® OS Integral therapy.

At baseline, findings indicated an anterior barycenter, reduced impact index (IMP), and left-side predominance.

**Figure 51:**
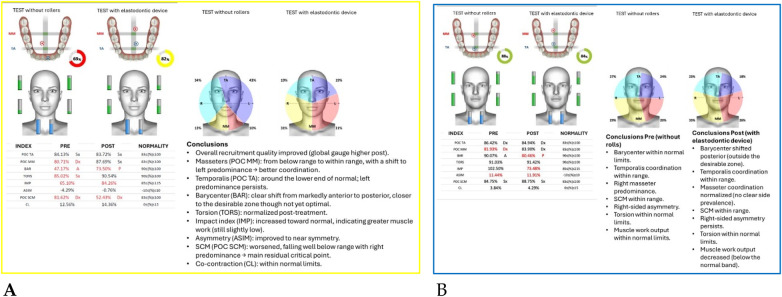
Teethan® surface electromyography. **(A)** Pre-treatment (without rollers); **(B)** post-treatment (with elastodontic device). After treatment, the barycenter shifted posteriorly, torsion normalized, and the impact index increased, indicating improved neuromuscular coordination and work efficiency.

After 14 months of treatment, muscle balance and coordination improved significantly, with normalization of barycenter position and functional indices (Table 26, Figures 51A,B).

**Table 26:** sEMG functional indices before and after AMCOP® OS integral therapy.

Index	Pre	Post	Outcome
POC TA	84.13%	86.42%	Within normal limits
POC MM	80.71%	81.93%	Stable within range
BAR	47.17%	90.07%	Normalized (anterior → central)
TORS	85.02%	91.03%	Normalized
IMP	65.10%	102.50%	From below → optimal
ASIM	−4.29%	11.44%	Within normal limits
POC SCM	81.62%	84.75%	Balanced
CL	12.56%	3.84%	Within range

#### 3.12.6 Interpretation

After 14 months of AMCOP® OS Integral therapy, the patient exhibited:

- Significant maxillary expansion across all reference points (Δ + 3.9–4.9 mm);
- Improved transverse and occlusal symmetry, particularly in the premolar and molar regions;
- Marked neuromuscular rebalancing, with normalization of barycenter (BAR ↑ from 47% → 90%) and IMP (↑ from 65% → 102%);
- Physiological muscle activation, indicating restoration of functional equilibrium.

The combination of morphometric and sEMG results confirms that AMCOP® OS Integral therapy effectively harmonized both skeletal and neuromuscular parameters in this early mixed dentition case.

## 4 Results

### 4.1 Sample and treatment overview

Twelve growing patients (8 females, 4 males; mean age 5.6 ± 0.8 years) completed treatment with AMCOP® elastodontic appliances. Therapy duration ranged from 10 to 18 months (per-protocol wear: 1 h/day + night-time use).

Patient selection was based exclusively on the presence of transverse maxillary deficiency; sagittal class was not considered an inclusion or exclusion criterion.

### 4.2 Intra-examiner reliability

Intra-examiner reliability analysis demonstrated excellent agreement for all transverse measurements. Intraclass correlation coefficient (ICC) values ranged from 0.92 to 0.98. Dahlberg's measurement error ranged between 0.18 and 0.32 mm. No repeated measurements exceeded the predefined tolerance threshold of 0.5 mm; therefore, no additional remeasurement or correction procedures were required.

### 4.3 Primary outcome: transverse changes on digital models

All patients exhibited an increase in transverse dimensions at the intercanine level, at the second deciduous molars (inter-5), and—when erupted—at the first permanent molars (inter-6). Aggregate data are summarized in Table 27.

**Table 27:** Transverse changes (Δpost–pre, mm) on digital models. Values expressed as mean ± SD (min–max); in parentheses: mean % variation vs. T0. Sample size per measurement: IC = 12; inter-5 = 12; inter-6 = 6.

Measure	Δ mm (mean ± SD) (range)	Mean % change	SMC (effect size)
Intercanine (IC)	+3.42 ± 1.52 (+0.50; +4.92)	+15.5%	2.25 (very large)
Inter-5 (second deciduous molars)	+4.11 ± 1.54 (+1.15; +6.26)	+15.6%	2.67 (very large)
Inter-6 (first molars)^a^	+3.34 ± 1.12 (+1.83; +4.87)	+11.3%	2.98 (very large)

a Inter-6 available in 6/12 cases (when permanent molars erupted). A sensitivity analysis restricted to the 6 patients with erupted first permanent molars showed transverse inter-6 increases consistent with those observed in the full sample, with a mean Δ of +3.34 ± 1.12 mm (range +1.83 to +4.87 mm), indicating that inclusion or exclusion of non-erupted molar cases did not materially affect the descriptive findings. SMC = standardized mean change calculated as mean change divided by standard deviation of the change scores. Effect interpretation based on Cohen's thresholds (0.2 small, 0.5 moderate, 0.8 large). Given the limited availability of inter-6 values, statistical description of transverse changes primarily relied on intercanine and inter-5 measures, which were available in all patients.

Post-hoc standardized mean change (SMC) effect sizes showed very large standardized effects for intercanine width (SMC = 2.25) and inter-5 width (SMC = 2.67). Inter-6 width, available in 6 patients only, also demonstrated a very large effect size (SMC = 2.98).

The transverse gain was generally homogeneous between anterior and posterior regions.

In cases treated with Class III (TC) devices, a slightly greater anterior expansion was observed, consistent with the functional design rationale, whereas Integral and OS devices showed a more harmonic or slightly posterior-dominant pattern. No reduction in transverse width was recorded in any patient. All repeated measurements fell within the pre-defined tolerance limit (≤0.5 mm). Data are presented descriptively without inferential statistical testing.measurements and sEMG indices for all patients are reported in Table 28.

**Table 28:** Individual anonymized transverse measurements and sEMG indices at T0 and T1.

Patient ID	IC T0	IC T1	Δ IC	Inter-5 T0	Inter-5 T1	Δ Inter-5	Inter-6 T0	Inter-6 T1	Δ Inter-6	BAR T0	BAR T1	POC-TA T0	POC-TA T1	POC-MM T0	POC-MM T1	TORS T0	TORS T1	ASIM T0	ASIM T1	IMP T0	IMP T1
P01	27.24	30.98	+3.74	30.60	34.60	+4.00	33.03	36.11	+3.08	41.31	92.09	81.17	88.68	81.17	85.57	87.17	92.23	16.49	5.26	23.78	103.27
P02	22.45	27.04	+4.59	29.14	34.02	+4.88	30.55	35.42	+4.87	85.12	90.62	85.41	86.33	56.81	88.39	79.57	90.53	28.89	0.55	134.58	88.59
P03	20.32	24.56	+4.24	25.38	28.70	+3.32	NA	NA	NA	85.12	90.62	85.41	86.33	56.81	88.39	79.57	90.53	28.89	0.55	134.58	88.59
P04	21.08	25.06	+3.98	24.69	30.95	+6.26	NA	NA	NA	23.74	90.00	78.74	87.26	89.73	83.00	91.52	90.50	7.41	0.00	26.31	85.00
P05	23.92	28.65	+4.73	29.01	35.19	+6.18	33.72	36.12	+2.40	66.99	68.54	75.35	84.84	83.50	88.93	90.52	91.67	−17.76	1.15	81.73	135.59
P06	23.35	24.33	+0.98	25.13	28.70	+3.57	NA	NA	NA	64.58	90.51	38.08	87.63	51.38	86.79	64.96	93.12	0.40	7.21	71.79	140.42
P07	23.22	24.71	+1.49	26.82	27.97	+1.15	27.51	29.34	+1.83	59.33	88.94	77.89	75.47	86.27	85.96	83.20	89.17	8.45	−14.54	50.09	59.14
P08	21.99	26.91	+4.92	27.46	31.60	+4.14	NA	NA	NA	75.39	90.62	61.78	82.40	61.64	86.06	75.69	92.17	−23.60	−14.17	164.42	113.94
P09	22.74	23.24	+0.50	27.35	29.51	+2.16	NA	NA	NA	55.56	87.42	85.19	75.06	84.58	73.04	88.39	92.50	4.46	25.78	68.81	101.09
P10	19.17	22.88	+3.71	25.79	29.15	+3.36	NA	NA	NA	64.58	87.17	38.08	80.51	51.38	82.29	64.96	83.66	0.40	0.99	71.79	317.28
P11	20.50	24.72	+4.22	22.52	27.95	+5.43	25.26	29.20	+3.94	41.31	91.90	81.17	83.79	81.17	86.85	87.17	89.83	16.49	3.06	23.78	95.63
P12	21.97	25.86	+3.89	26.30	31.21	+4.91	29.59	33.49	+3.90	47.17	90.07	84.13	86.42	80.71	81.93	85.02	91.03	−4.29	11.44	65.10	102.50

Inter-6 measurements were available only for patients with erupted first permanent molars (*n* = 6).

BAR, POC TA/MM, TORS, ASIM, and IMP values expressed according to Teethan® system indices. Patients were anonymized according to institutional privacy regulations. Only variables explicitly available from case records and reported in the manuscript are included.

Individual transverse

Missing cells indicate unavailable or non-recorded data (e.g., unerupted molars or incomplete sEMG documentation).

### 4.4 Secondary outcomes: neuromuscular balance (Teethan® sEMG)

Pre- and post-treatment surface electromyography (sEMG) analysis demonstrated systematic improvement across all functional indices:

- Barycenter (BAR): normalization toward the desired zone in 11/12 patients; one case showed improvement without full normalization but with better recruitment quality.
- POC TA/MM: transition from below-range to within-range values in most cases, particularly in patients with more pronounced baseline asymmetry; in the remaining cases, values stayed within the physiological range with improved bilateral coordination.
- Torsion (TORS): reduction or normalization of torsion in > 80% of patients.
- Global asymmetry (ASIM): decreased in 10/12 cases, returning to symmetry or physiological limits.
- Impact/Co-contraction (IMP/CL): increased muscular efficiency (higher IMP) in most cases, with CL values remaining within the normal range.

Overall, post-treatment traces described neuromuscular rebalancing consistently with the transverse expansion documented on 3D models. *post-hoc* standardized mean change (SMC) effect sizes were calculated for the primary transverse outcomes using the mean change divided by the standard deviation of change scores. Very large standardized effects were observed for intercanine width (SMC = 2.25) and inter-5 width (SMC = 2.67). Inter-6 width, available in 6 patients, also showed a very large effect size (SMC = 2.98).

Aggregated pre- and post-treatment values for all sEMG functional indices, including variability ranges and normalization rates, are summarized in Table 29.

**Table 29:** Aggregated surface electromyography (sEMG) indices before (T0) and after treatment (T1). Values expressed as mean ± SD (range). “normalized” indicates achievement of accepted physiological thresholds at T1.

Index	T0 Mean ± SD (range)	T1 Mean ± SD (range)	Normalized at T1 *n* (%)
POC TA (%)	72.8 ± 18.6 (38.1–85.4)	83.2 ± 4.5 (75.1–88.7)	9/12 (75%)
POC MM (%)	72.2 ± 15.3 (51.4–89.7)	84.8 ± 4.5 (73.0–88.9)	10/12 (83%)
BAR (%)	59.2 ± 19.0 (23.7–85.1)	88.9 ± 2.7 (87.2–92.1)	11/12 (92%)
TORS (%)	81.9 ± 9.4 (65.0–91.5)	90.9 ± 3.2 (83.7–93.1)	11/12 (92%)
ASIM (%)	5.4 ± 16.2 (−23.6 – + 28.9)	2.0 ± 11.2 (−14.5 – + 25.8)	10/12 (83%)
IMP (%)	83.9 ± 42.6 (23.8–164.4)	120.8 ± 66.5 (59.1–317.3)	9/12 (75%)
POC SCM (%)	81.3 ± 2.7 (78.9–83.9)	82.8 ± 4.7 (72.7–89.9)	9/12 (75%)

### 4.5 Radiographic overview and clinical course

Pre- and post-treatment panoramic radiographs were obtained to document dental eruption status but were not used for transverse measurements. Overall, they showed symmetric eruption patterns and absence of pathological findings. Adverse events were actively monitored throughout the treatment period and at each scheduled follow-up visit (every 4–6 weeks). At every appointment, parents were questioned and children were clinically examined for the occurrence of potential side effects, including soft tissue irritation or ulceration, speech or swallowing discomfort, feeding difficulties, abnormal tooth mobility, dental pain, or appliance intolerance. Panoramic radiographs obtained for routine monitoring were also inspected for signs of pathological root changes. No additional follow-up beyond the active treatment period was available.

### 4.6 Individual case highlights

The twelve individual cases are detailed in the *Case Series* section, including pre-/post-palatal 3D scans, digital bite models, and pre-/post-sEMG reports. In summary:

- Intercanine expansion: ↑ in 12/12 cases (+0.5 – + 4.9 mm);
- Inter-5 expansion: ↑ in 12/12 cases (+1.15 – + 6.26 mm);
- Inter-6 expansion (measurable in 6 cases): ↑ in 6/6 cases (+1.83 – + 4.87 mm).

### 4.7 Intra-examiner reliability

Intra-examiner reliability was assessed on a random 20% subsample of models remeasured after 7 days.

Excellent agreement was observed for all transverse measurements.

The intraclass correlation coefficient (ICC) values were:

- Intercanine width: ICC = 0.94 (95% CI: 0.90–0.98)
- Inter–second deciduous molar width (inter-5): ICC = 0.92 (95% CI: 0.88–0.97)
- Inter–first molar width (inter-6, when available): ICC = 0.98 (95% CI: 0.95–0.99)

Dahlberg's method error ranged between 0.18 and 0.32 mm across all measurements.

No discrepancies exceeded the pre-defined tolerance threshold of 0.5 mm.

Accordingly, measurement reliability was considered clinically acceptable for all evaluated levels.

## 5 Discussion

Given the retrospective case-series design, the findings describe associations rather than causal effects. The observed transverse improvements and neuromuscular changes should therefore be interpreted as exploratory trends within this sample, without assuming treatment efficacy. Future controlled and randomized studies are necessary to confirm causal relationships.

### 5.1 Main findings and clinical interpretation

Early orthodontic intervention plays a decisive role in the management of malocclusions and associated orofacial dysfunctions. The present case series suggests that AMCOP® elastodontic appliances are associated with physiological transverse development in growing patients, with consistent transverse and neuromuscular improvement documented through digital models and sEMG. The protocol adopted in this study, beginning with the Integral masticatory plane to correct transverse and vertical dimensions, followed by class-specific devices for sagittal refinement—proved clinically successful (117–121). Digital model analysis confirmed significant increases in intercanine, inter-premolar, and intermolar widths, while sEMG traces (Teethan®) documented restoration of muscular symmetry, barycenter normalization, and improved coordination between the temporalis and masseter muscles. The functional design of AMCOP®, characterized by an elastic thermoplastic blend and dedicated occlusal planes, allows the appliance to work simultaneously on the skeletal base, dental elements, and musculature, promoting stable orthopedic and neuromuscular remodeling. The use of different AMCOP® designs (Integral S/OS and TC) reflects individualized treatment needs and introduces some heterogeneity in the intervention, which may partly explain the variability in clinical and sEMG responses. Importantly, the results were obtained without the need for adjunctive myofunctional exercises, since the natural swallowing reflex ensured continuous functional activation during use.

### 5.2 Comparison with existing concepts and clinical approaches

The present findings align with the established understanding that traditional expansion appliances, such as Hyrax, Haas, or quad-helix, are primarily mechanical devices designed to achieve rapid transverse widening through orthopedic forces. While effective in separating the midpalatal suture, these appliances do not directly address the neuromuscular and postural components that influence long-term stability (122–128).

Functional appliances such as Activator or Twin Block mainly focus on sagittal correction and show limited influence on the transverse plane. In contrast, the AMCOP® system operates through gentle elastic stimulation that integrates skeletal, dental, and muscular adaptation within a single protocol. This approach, as emphasized in previous conceptual frameworks of functional orthopedics, prioritizes tongue posture normalization, balanced muscle recruitment, and physiologic guidance of craniofacial growth.

The transverse increases observed in our cases (average 3–5 mm) are consistent with what can be expected from gradual functional expansion during early mixed dentition (129–135). The concurrent improvement in electromyographic symmetry supports the notion that skeletal correction and muscular rebalancing can occur simultaneously when treatment is started early and conducted under functional control. Overall, these results strengthen the rationale for elastodontic and biofunctional methods as complementary tools in the modern management of transverse maxillary deficiencies (136–144).

### 5.3 Clinical implications

The presented protocol underlines the importance of addressing transverse deficiency before sagittal correction. Treating the transverse plane first ensures that subsequent class-oriented elastodontic therapy (e.g., Class III or II AMCOP®) acts on a balanced and coordinated craniofacial base. Failure to correct the transverse component beforehand is one of the most frequent causes of suboptimal outcomes in functional orthopedic therapy. AMCOP® therapy was well tolerated, with high compliance thanks to its non-invasive design, passive nocturnal use, and minimal discomfort. The versatility of the system allows adaptation to individual growth stages, providing a flexible, phase-oriented approach suitable for both children and adults (12, 145–154).

### 5.4 Limitations and future perspectives

This study is limited by its case-series design, small sample size, and absence of a control group, which precludes inferential statistical conclusions. Furthermore, no orthopedic or orthodontic retention appliances were prescribed after the active phase, and no standardized post-treatment follow-up beyond the end-of-treatment evaluation was performed to assess stability or relapse; therefore, the lack of long-term follow-up does not allow assessment of post-treatment stability. Future research should include prospective, controlled trials comparing AMCOP® with conventional expansion protocols using randomized allocation or matched cohort designs and integrating three-dimensional CBCT analysis with sEMG and postural assessments to further characterize the anatomical correlates of the transverse dimensional changes observed. The present findings describe transverse skeletal changes and neuromuscular pattern variations observed following AMCOP® elastodontic therapy in growing patients. The device features, including functional mechanism, comfort, and adaptability, support its consideration as a potential early interceptive option for the management of transverse maxillary deficiencies and related functional disturbances, pending confirmation from controlled clinical studies (155–157). Surface EMG in pediatric patients presents inherent variability due to cooperation, immature neuromotor patterns, difficulty maintaining standardized occlusal posture, and behavioral fluctuations. These factors may influence signal stability and must be considered when interpreting sEMG changes. Another limitation of this study concerns the non-uniform orientation of some digital occlusal images, which were retrieved from archived clinical material and could not be fully standardized due to lack of access to original raw data. In addition, treatment compliance was assessed subjectively through parental reporting rather than by objective wear-time monitoring systems, which may have introduced reporting bias. Since no CBCT or radiographic angular measurements were performed, the present investigation cannot characterize the biological nature of the transverse dimensional changes observed on digital dental models. Furthermore, part of the transverse increases observed may be attributable to physiological craniofacial growth and dental eruption expected in the 4–7 year age range. Longitudinal growth studies report spontaneous transverse maxillary increases in untreated children of approximately 0.5–1.0 mm per year at the intercanine and intermolar levels during early mixed dentition, depending on sex and developmental stage. Therefore, without an untreated control group, it is not possible to quantify precisely the relative contributions of natural growth vs. appliance-induced orthopedic and dentoalveolar effects. Additionally, the absence of examiner blinding may have introduced a potential measurement bias, although high intra-examiner reliability values reduce this concern. From a clinical standpoint, these findings support the early use of elastodontic functional appliances as a conservative interceptive approach for transverse deficiencies in growing patients. Early interception may contribute to restoring both skeletal dimensions and neuromuscular balance during phases of high biological plasticity, potentially reducing the need for more invasive orthopedic or orthodontic procedures in later stages. Moreover, the combined use of digital model analysis and sEMG monitoring may provide clinicians with an objective tool to evaluate functional adaptation throughout treatment.

## 6 Conclusions

The present study confirms that AMCOP® elastodontic appliances represent a valid and innovative approach for the early correction of transverse maxillary deficiencies in growing patients. Digital model measurements and surface electromyographic analyses showed transverse dimensional changes and neuromuscular pattern variations associated with the use of the bioactivators. The observed improvements in transverse width and in muscle symmetry parameters (barycenter, torsion, and impact indices) highlight the transverse and neuromuscular changes observed in patients treated with AMCOP® devices, which were characterized by more balanced transverse dimensions and improved neuromuscular indices, although causal inferences cannot be drawn. Compared with traditional mechanical expanders, the AMCOP® system provides a more comfortable, compliant, and biologically oriented therapy, particularly suitable for the interceptive phase of craniofacial growth. From a scientific perspective, these findings contribute to the growing evidence supporting elastodontic approaches as a valuable frontier in functional orthodontics. Given the limited sample size and the observational case-series design, the present findings refer exclusively to the investigated cases and cannot be generalized to the broader pediatric population. Further controlled studies with larger samples and long-term follow-up are encouraged to consolidate these preliminary results and to deepen the understanding of the interplay between skeletal, muscular, and postural components during growth.

## Data Availability

The datasets presented in this study can be found in online repositories. The names of the repository/repositories and accession number(s) can be found in the article/supplementary material.
